# Calcineurin complex isolated from T-cell acute lymphoblastic leukemia (T-ALL) cells identifies new signaling pathways including mTOR/AKT/S6K whose inhibition synergize with calcineurin inhibition to promote T-ALL cell death

**DOI:** 10.18632/oncotarget.9933

**Published:** 2016-06-10

**Authors:** Valeria Tosello, Valentina Saccomani, Jiyang Yu, Fulvio Bordin, Alberto Amadori, Erich Piovan

**Affiliations:** ^1^ UOC Immunologia e Diagnostica Molecolare Oncologica, Istituto Oncologico Veneto-IRCCS, Padova, 35128, Italy; ^2^ Dipartimento di Scienze Chirurgiche, Oncologiche e Gastroenterologiche, Sezione di Oncologia, Universita' di Padova, Padova, 35128, Italy; ^3^ Department of Biomedical Informatics, Columbia University, New York, NY, 10032, USA; ^4^ Department of Systems Biology, Columbia University, New York, NY, 10032, USA; ^5^ Present address: Department of Precision Medicine, Oncology Research Unit, Pfizer Inc., Pearl River, NY, 10965, USA

**Keywords:** calcineurin, T-cell acute lymphoblastic leukemia, mTOR signaling, apoptosis, protein complex

## Abstract

Calcineurin (Cn) is a calcium activated protein phosphatase involved in many aspects of normal T cell physiology, however the role of Cn and/or its downstream targets in leukemogenesis are still ill-defined. In order to identify putative downstream targets/effectors involved in the pro-oncogenic activity of Cn in T-cell acute lymphoblastic leukemia (T-ALL) we used tandem affinity chromatography, followed by mass spectrometry to purify novel Cn-interacting partners. We found the Cn-interacting proteins to be part of numerous cellular signaling pathways including eIF2 signaling and mTOR signaling. Coherently, modulation of Cn activity in T-ALL cells determined alterations in the phosphorylation status of key molecules implicated in protein translation such as eIF-2α and ribosomal protein S6. Joint targeting of PI3K-mTOR, eIF-2α and 14-3-3 signaling pathways with Cn unveiled novel synergistic pro-apoptotic drug combinations. Further analysis disclosed that the synergistic interaction between PI3K-mTOR and Cn inhibitors was prevalently due to AKT inhibition. Finally, we showed that the synergistic pro-apoptotic response determined by jointly targeting AKT and Cn pathways was linked to down-modulation of key anti-apoptotic proteins including Mcl-1, Claspin and XIAP. In conclusion, we identify AKT inhibition as a novel promising drug combination to potentiate the pro-apoptotic effects of Cn inhibitors.

## INTRODUCTION

T-cell acute lymphoblastic leukemia (T-ALL) accounts for approximately 15% of pediatric and 25% of adult cases of ALL. Progress in T-ALL therapy has been impressive, with cure rates approaching 80% for children and 50% for adults [1, 2], however only limited therapeutic options are available for patients with primary resistant or relapsed disease. T-ALL is classified into subgroups based on exclusive genetic alterations and/or deregulated expression of specific transcription factors, including the TALL1/2, LMO1/2, TLX1/3 and HOXA families, associated with distinct stages of differentiation arrest [3-5].

Calcineurin (PPP3; PP2B referred to as Cn) is a calcium-activated serine/threonine (S/T) phosphatase, composed of a catalytic subunit (PPP3CA) and a regulatory subunit (PPP3CB), critical to a number of developmental processes in the cardiovascular, nervous, and immune system. Cn has the ability to dephosphorylate a broad range of proteins, including Nuclear Factor of Activated T cells (NFAT) proteins. Although critically involved in many aspects of normal T cell survival, proliferation and activation, the direct implication of Cn and/or its downstream NFAT targets in lymphomagenesis and cancer in general has only recently been reported [6, 7]. Available evidence shows that NFAT transcription factors are mediators of Cn action in different cancers [8-10]. However, it is possible that NFAT factors are not the only targets of Cn in leukemogenesis, as Cn can dephosphorylate other factors possibly relevant to its oncogenic properties. With this perspective in mind, we used tandem affinity chromatography, followed by mass spectrometry (MS) to purify novel Cn-interacting partners in human T-ALL cells. We found a large set of proteins associated with Cn, including transcriptional regulators and protein modifiers which fell into numerous canonical signaling pathways. In this study, we explored the potential therapeutic importance of the functional interaction between the top signaling pathways enriched in our PPP3CA complex and the canonical Cn-NFAT signaling pathway.

## RESULTS

### Identification of PPP3CA–associated binding partners in T-ALL cells

**Figure 1:**
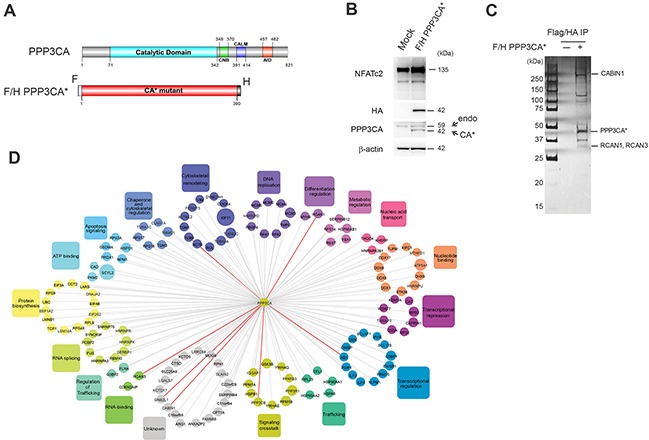
Affinity purification mass spectrometry identifies PPP3CA-associated proteins **A.** Schematic representation of the functional domains of PPP3CA (top panel) including the catalytic domain, the CnB binding region (CNB), the calmodulin binding region (CALM) and the autoinhibitory domain (AID). Schematic representation of the constitutively active mutant (PPP3CA*) containing FLAG (F) and HA epitope (H) tags. This mutant lacks the calmodulin binding and autoinhibitory domains (bottom panel). Numbers indicate relative amino acid residues. **B.** Expression level of FLAG/HA epitope-tagged PPP3CA constitutively active mutant (F/H-PPP3CA*) in Jurkat whole cell extracts was measured by Western blot using an anti-HA antibody that recognizes only exogenous tagged PPP3CA (CA*) and anti-PPP3CA antibody that recognizes both endogenous (endo) and tagged PPP3CA (CA*). NFATc2 expression was used as a surrogate of Cn activation. β-actin was used as loading control. **C.** Purification of PPP3CA and its interacting partners. Whole cell extracts prepared from F/H-PPP3CA*-expressing Jurkat cells or mock-transduced (−) Jurkat cells were subjected to sequential immunoprecipitation using anti-FLAG and anti-HA beads. Proteins were resolved by SDS-PAGE and visualized by silver staining. Molecular weights are indicated on the left. Some known and identified interacting proteins are shown on the right. **D.** Interaction network of PPP3CA-associated proteins identified by mass spectrometry. The false positive interactors were excluded by removing all proteins that were also detected in the control purification. Known identified interacting proteins are shown with bold red lines.

To identify novel factors that support the putative oncogenic function of Cn in T-ALL, we purified activated PPP3CA from Jurkat T-ALL cells. To this end, we generated Jurkat cells stably expressing a human constitutively active PPP3CA mutant (PPP3CA*) tagged with both FLAG and HA epitopes (F/H-PPP3CA*). This mutant lacks both the calmodulin binding domain and auto-inhibitory domains and is constitutively active [11] even in the absence of elevated calcium (Figure 1A). Immunoblot analysis showed that tagged PPP3CA* is expressed at a level comparable to endogenous PPP3CA (Figure 1B). Importantly, F/H-PPP3CA* expressing cells had higher levels of NFATc2 and dephosphorylated (fast migrating in SDS/PAGE) forms of NFATc2 compared to empty vector-expressing control cells (Figure 1B). F/H-PPP3CA* and its associated partners were purified from total cell lysates derived from Jurkat cells using tandem affinity chromatography (Figure 1C). Mass spectrometry analysis identified 139 protein-partners of PPP3CA* (Figure 1D and Supplementary Table S1). Importantly, numerous well-characterized PPP3CA-interacting proteins were identified in our complex (Figure 1D and Supplementary Table S1), including PPP3R1, RCAN1 and 3, PPP3CB, CABIN1 and IQGAP1 [12]. Interaction network analysis of PPP3CA*-associated proteins revealed several functional classes of proteins including proteins with transcriptional regulatory activity, enzyme regulatory activity and translational regulatory activity (Figure 1D). Interestingly, several proteins implicated in leukemia pathobiology emerged amongst the novel PPP3CA interactors such as NPM1, BCL11b, GSK3β and KDM1 (also known as LSD1). Amongst the most prominent functional categories identified by Database for Annotation, Visualization and Integrated Discovery (DAVID) were proteins involved in apoptosis signaling, transcriptional regulation/repression and signaling crosstalk (Supplementary Table S1). Similarly, functional classification using PANTHER bio-informatics tool identified numerous functional categories to be enriched in the protein list including chaperone activity, binding activity, transcriptional regulator activity and enzyme regulator activity (Supplementary Figure S1). STRING program was also used to generate a protein interaction network from our identified PPP3CA interacting proteins integrating interaction data from numerous sources including genomic context, high-throughput experiments, co-expression experiments and previous knowledge from the literature (Supplementary Figure S1).

### Validation of novel identified PPP3CA interacting proteins

**Figure 2:**
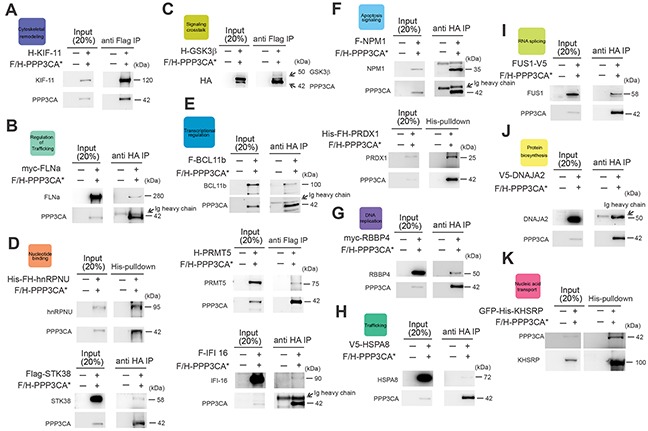
Validation of novel PPP3CA interacting proteins HEK 293T cells were transfected with FLAG/HA epitope-tagged PPP3CA* mutant (F/H-PPP3CA*) and expression plasmids for each of the identified PPP3CA-interacting proteins. Western blot analysis of **A.** KIF-11 (anti-HA), **B.** FLNa (anti-myc), **C.** GSK3β (anti-HA), **D.** (lower panel) STK38 (anti-FLAG), **E.** (upper panel) BCL11b (anti-FLAG), (**E**), (middle panel) PRMT5 (anti-HA), (**E**), (lower panel) IFI-16 (anti-FLAG), **F.** (upper panel) NPM1 (anti-FLAG), **G.** RBBP4 (anti-myc), **H.** HSPA8 (anti-V5), **I.** FUS1 (anti-V5), **J.** DNAJA2 (anti-V5) after PPP3CA* immunoprecipitation with anti-FLAG or anti-HA beads in HEK 293T cells is shown. HEK 293T (FH-His) cells were transfected with F/H PPP3CA* mutant and expression plasmids for FLAG-HA-Histidine tagged (F/H-His) hnRNPU or PRDX1. (**D**, upper panel) Western blot analysis of PPP3CA* and hnRNPU (anti-HA) or (**F**, lower panel) PPP3CA* and PRDX1 (anti-FLAG) after His-pulldown in HEK 293T cells. **K.** HEK 293T cells were transfected with F/H-PPP3CA* and expression plasmids for Green Fluorescent Protein (GFP) and Histidine-tagged (His) KHSRP. Immunoblot analysis of PPP3CA* (anti-FLAG) after His-pulldown in HEK 293T cells expressing F/H-PPP3CA* and His-tagged GFP-KHSRP is shown.

**Figure 3:**
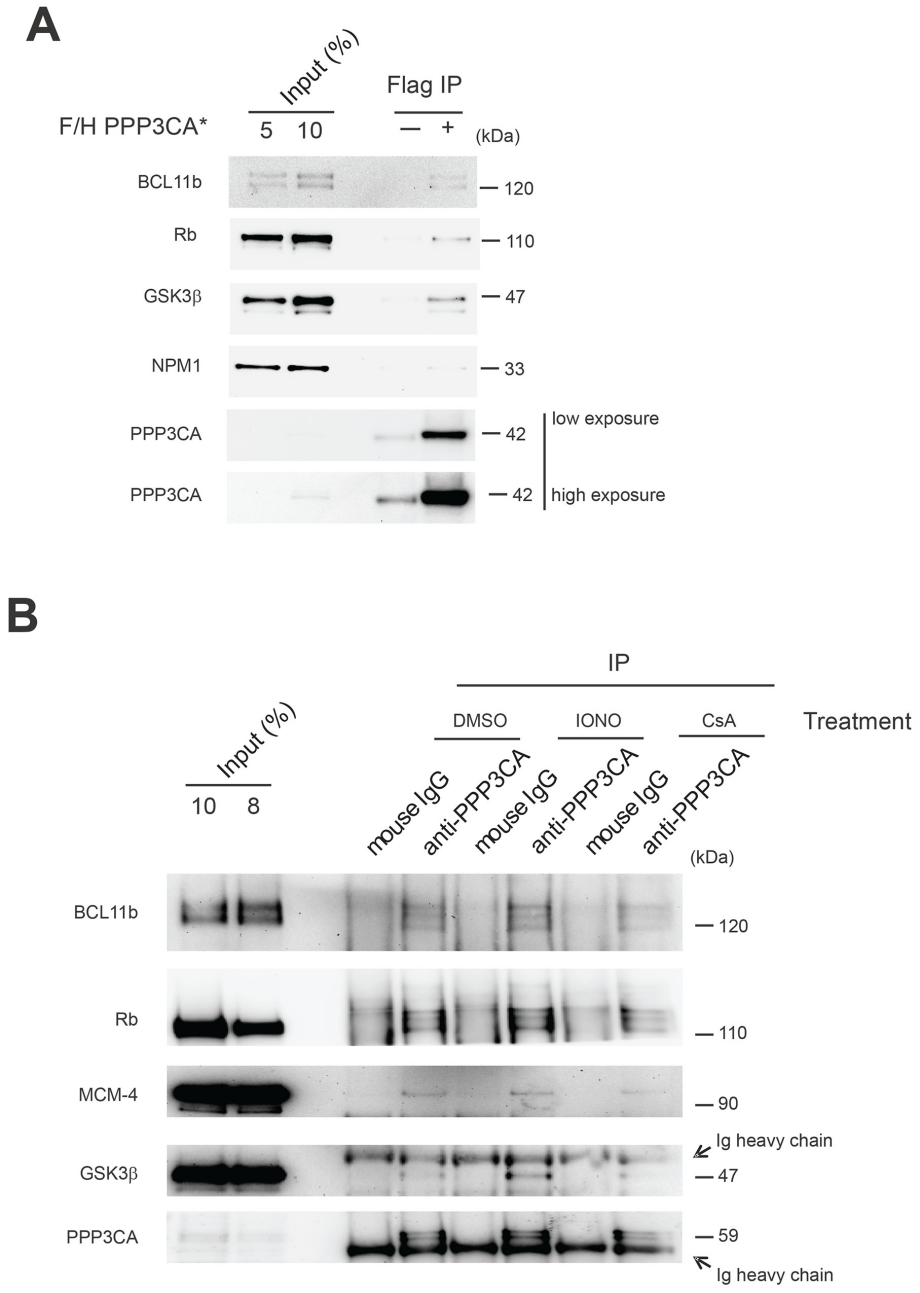
Validation of selected PPP3CA-interacting proteins in Jurkat T-ALL cells **A.** Immunoblot analysis of BCL11b, Rb, GSK3β and NPM1 after PPP3CA protein immunoprecipitation (IP anti-FLAG) in F/H-PPP3CA*-expressing Jurkat cells or mock-transduced (−) Jurkat cells. **B.** Analysis of protein interactions by immunoblot analysis of BCL11b, Rb, MCM-4 and GSK3β after PPP3CA protein immunoprecipitation under basal conditions and after treatment with Ionomycin (IONO) or cyclosporine (CsA) in Jurkat T-ALL cells.

To validate our results, proteins belonging to the major DAVID functional annotation clusters identified and distributed relatively homogeneously in our ordered protein list (Supplementary Table S1) were cloned and tested in co-transfection experiments. In these experiments HEK 293T cells were transfected with constitutively active PPP3CA* and each of the putative interacting proteins belonging to the following functional categories: cytoskeletal remodeling (KIF11), regulation of trafficking (FLNA), signaling crosstalk (GSK3β), nucleotide binding (hnRNPU, STK38), transcriptional regulation (PRMT5, IFI16, BCL11b), ATP binding (PKM2), apoptosis signaling (NPM1, PRDX1), DNA replication (RPA2, RBBP4), trafficking (HSPA8), RNA splicing (FUS), nucleic acid transport (KHSRP) and protein biosynthesis (DNAJA2). In all, after immunoprecipitation (IP), we confirmed 15 of the 17 tested putative interacting proteins to be able to interact with PPP3CA (>90%; Figure 2). Some of the PPP3CA interactor proteins implicated in leukemia pathobiology such as BCL11b, NPM1, GSK3β and Rb were further validated in Jurkat T-ALL cells expressing constitutively active PPP3CA* (Figure 3A). To study whether some of these interactions were also present in T-ALL cells under endogenous conditions, we lysed Jurkat T-ALL cells and executed IP against PPP3CA and subsequently immunoblotted against BCL11b, Rb, MCM-4 and GSK3β (Figure 3B). In addition, to explore further the functional relevance of the interactions we executed IP following treatment with ionomycin (IONO; to activate Cn/NFAT signaling), Cyclosporin A (CsA; to inactivate Cn/NFAT signaling) or vehicle control for 1h, in order to modulate Cn activity. We found that the interaction between PPP3CA and BCL11b, Rb and GSK3β present under basal conditions was further increased following acute stimulation of Cn by ionomycin, especially for GSK3β (as previously reported in transfected cells [13]). On the other hand, CsA treatment did not significantly modify the basal interaction with these proteins.

### EIF2 and mTOR/p70S6K signaling pathways identified through analysis of canonical signaling pathways enriched in the PPP3CA-binding proteins isolated from T-ALL are altered following modulation of Cn activity

**Figure 4:**
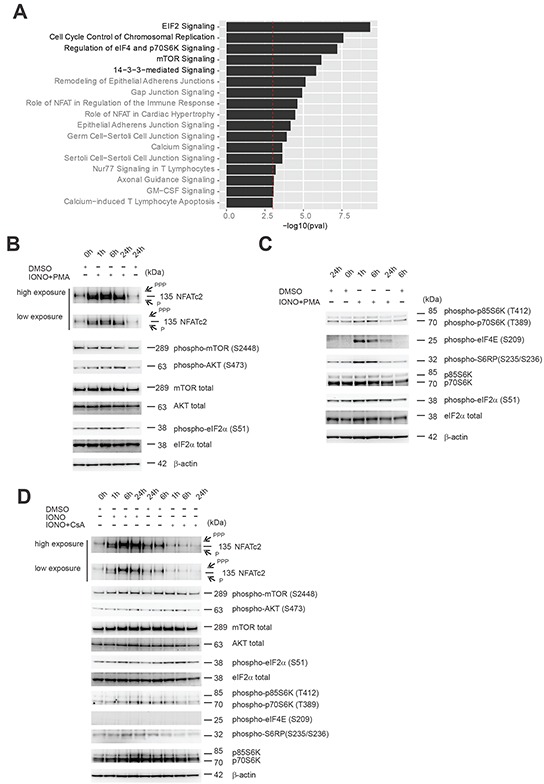
Effects of Cn activity modulation on components of eIF2 and mTOR signaling pathways found to be enriched in PPP3CA-binding proteins **A.** Ingenuity software was used to assess for enrichment of specific pathways using the identified protein list of PPP3CA binding proteins. All pathways that demonstrated enrichment with –log (*P* value) >3 are shown and ordered based on the *P* value. **B.** Western blot analysis of NFATc2, phospho-mTOR (S2448), phospho-AKT (S473), phospho-eIF2α (S51) expression in Jurkat T-ALL cells treated for different times (0, 1, 6, 24h) with vehicle only or the combination Ionomycin (IONO) and Phorbol myristate acetate (PMA) (0.5μg/mL and 100ng/mL, respectively). mTOR, AKT, eIF2α and β-actin are shown as loading controls. **C.** Western blot analysis of phospho-p70/p85 S6K (T389/T412), phospho-eIF4E (S209), phospho-S6RP (S235/236), phospho-eIF2α (S51), p70/p85 S6K expression in Jurkat T-ALL cells treated for different times (0, 1, 6, 24h) with vehicle only or the combination Ionomycin (IONO) and Phorbol myristate acetate (PMA) (0.5μg/mL and 100ng/mL, respectively). eIF2α and β-actin are shown as loading controls. **D.** Western blot analysis of NFATc2, phospho-mTOR (S2448), phospho-AKT (S473), phospho-eIF2α (S51), phospho-p70/p85 S6K (T389/T412), phospho-eIF4E (S209), phospho-S6RP (S235/236), p70/p85 S6K expression in Jurkat T-ALL cells treated for different times (0, 1, 6, 24h) with vehicle only, Ionomycin (IONO) or CsA (0.5μg/mL and 10μg/mL, respectively). mTOR, AKT, eIF2ξ03B1; and β-actin are shown as loading controls. PPP=hyper-phosphorylated NFATc2; P=hypo-phosphorylated NFATc2.

To identify signaling pathways enriched in the identified PPP3CA-binding partners we made use of the Ingenuity pathways analysis software (Qiagen, Germantown, MD, USA). The top canonical signaling pathways are shown in Figure 4A, and included eIF2 signaling, cell cycle control, mammalian target of Rapamycin (mTOR) signaling and 14-3-3 mediated signaling. The complete list of canonical signaling pathways enriched in our protein list is show in Supplementary Table S2. Within this list several well-known pathways associated with NFAT-Cn function such as role of NFAT in regulation of the immune system, role of NFAT in cardiac hypertrophy, calcium signaling and calcium-induced T lymphocyte apoptosis were significantly enriched (Figure 4A). To explore the functional relevance of these findings, we performed experiments to determine the effects of Cn-NFAT signaling activation and inhibition on eIF2/p70S6K and PI3K/AKT/mTOR signaling pathways. To this end, Jurkat T-ALL cells were treated for different times with the combination ionomycin and the phorbol ester PMA (Phorbol myristate acetate; IONO+PMA; to induce optimal Cn-NFAT activation). As shown in Figure 4B, Cn activation determined a moderate increase in phosphorylated AKT (phospho-AKT; S473) and phospho-eIF2α (S51), while no significant modulation of phospho-mTOR (S2448) was found. In addition, Cn activation determined a significant increase in phospho-S6K (T389), phospho-S6RP (S235/236) and phospho-eIF4E (S209), especially at early time points (Figure 4C). Phosphorylation of eIF2α is implicated in inhibition of eIF2B activity, while phosphorylation of eIF4 and S6RP are indicative of increased protein synthesis, thus it appears that the relationship between Cn activation and protein synthesis is rather complex. Possibly, acute activation of Cn may lead to a transient increase in mRNA translation and protein synthesis, while prolonged Cn activation ultimately results in a decrease in protein synthesis. Activation of Cn by ionomycin alone (Figure 4D) also determined an increase in phospho-AKT (S473), phospho-eIF2α (S51), phospho-S6K (T389) and phospho-S6RP (S235/236), while it was ineffective in inducing phospho-eIF4E (S209). On the other hand, Cn inhibition by CsA had a mixed effect (Figure 4D) as it also increased phospho-AKT (S473), phospho-eIF2α (S51), while it reduced phospho-S6K (T389) and phospho-S6RP (S235/236). All together, these results indicate that Cn activity can significantly modulate signaling pathways implicated in mRNA translation and protein synthesis.

### Inhibition of PI3K-mTOR signaling in combination with Cn inhibition promotes T-ALL cell death in T-ALL cell lines

**Figure 5:**
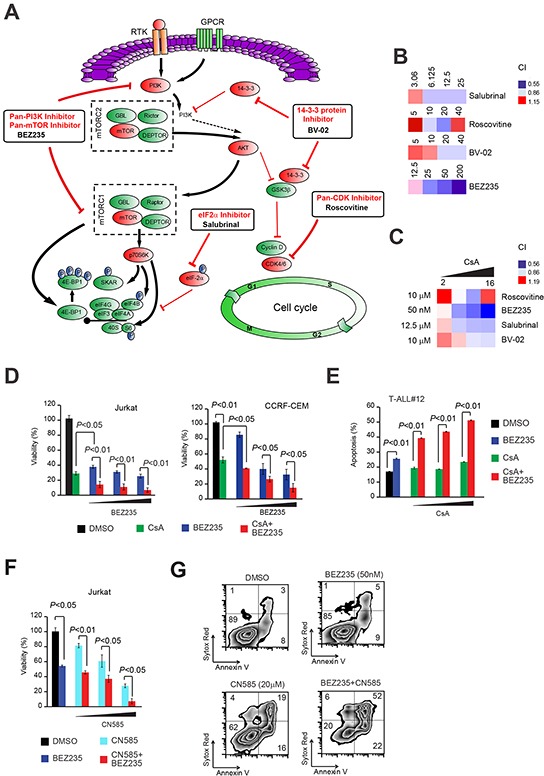
Joint pharmacologic inhibition of Cn with inhibitors of canonical pathways enriched in PPP3CA-binding proteins identifies PI3K-mTOR inhibition as the most synergistic anti-leukemic combination **A.** Schematic representation of signaling pathways found enriched in PPP3CA interacting proteins and their inhibitors. Pathway inhibition is shown in red. **B.** Heat map representation of combination indexes (CI) of tested pathway inhibitors (used at different concentrations; shown) with a fixed concentration of the Cn inhibitor CsA (8μg/mL) in Jurkat T-ALL cells. Concentrations are in the nano Molar (nM) range for BEZ235, while are in the micro-Molar (μM) range for all other inhibitors. CI>1.1 indicates antagonism, CI<1 indicates synergism. **C.** Heat map representation of combination indexes (CI) of tested pathway inhibitors (used at a fixed dose, shown) with different concentrations of the Cn inhibitor CsA (2, 4, 8, 16 μg/mL) in Jurkat T-ALL cells. **D.** (left panel) Cell viability quantification in Jurkat T-ALL cells treated *in vitro* with vehicle only, BEZ235 (50-500 nM), CsA (10 μg/mL) or CsA plus BEZ235 (CsA + BEZ235; 10 μg/mL and 50-500 nM, respectively) for 72h. Error bars represent ± SD of triplicate experiments. (**D**, right panel) Cell viability quantification in CCRF-CEM T-ALL cells treated *in vitro* with vehicle only, BEZ235 (20-100 nM), CsA (10 μg/mL) or CsA plus BEZ235 (CsA + BEZ235; 10 μg/mL and 20-100 nM, respectively) for 72h. Error bars represent ± SD of triplicate experiments. **E.** Apoptosis quantification in T-ALL xenograft #12 (T-ALL#12) cells treated *in vitro* with vehicle only, BEZ235 (2 μM), CsA (2.5-10 μg/mL) or BEZ235 plus CsA (BEZ235 + CsA; 2 μM and 2.5-10 μg/mL, respectively). Error bars represent ± SD of triplicate experiments. **F.** Cell viability quantification in Jurkat T-ALL cells treated *in vitro* with vehicle only, BEZ235 (50 nM), CN585 (10-20 μM) or BEZ235 plus CN585 (BEZ235 + CN585; 50nM and 10-20 μM, respectively) for 72h. Error bars represent ± SD of triplicate experiments. **G.** Representative plots of apoptosis in Jurkat T-ALL cells treated *in vitro* for 48h with vehicle only, BEZ235 (50 nM), CN585 (20 μM) or CN585 plus BEZ235 (CN585 + BEZ235; 20 μM and 50 nM, respectively).

Persistent Cn/NFAT signaling has been shown to be pro-oncogenic *in vivo* in mouse models of human T-ALL/lymphoma [14] and very recently Cn has been shown to be essential for the ability of T-ALL leukemic cells to long-term propagate the disease in serial transplantation assays [15]. Since several of the signaling pathways found enriched in our complex are aberrantly activated or deregulated in T-ALL and pharmacological inhibitors to some of the enriched canonical pathways exist, we evaluated whether a functional interaction between the top signaling pathways enriched in our PPP3CA complex and the canonical PPP3CA-NFAT signaling pathway existed. Thus, we evaluated whether pharmacological inhibition of the pathways: (i) cell cycle control (using the pan-CDK inhibitor, Roscovitine), (ii) mTOR signaling (using the PI3K-mTOR inhibitor, BEZ235), (iii) eIF2 signaling (using the eIF2α inhibitor, Salubrinal) and (iv) 14-3-3 signaling (using BV-02) could be potentially exploited therapeutically in T-ALL in combination with Cn inhibitors such as CsA and/or other Cn specific inhibitors such as CN585 [16] or FK-506. To this end, Jurkat T-ALL cells were treated with increasing concentrations of each of the afore mentioned pathway inhibitors (Roscovitine, BEZ235, Salubrinal or BV-02) or vehicle in combination with the Cn inhibitor, CsA and evaluated for loss of viability. Analysis of drug interactions using the median-effect method of Chou and Talay [17] to calculate the combination index (CI), disclosed predominantly a synergistic anti-leukemic effect in the combination CsA and Salubrinal, BV-02 and BEZ235 [CI<1] at multiple concentrations (Figure 5B and 5C). Of these, the PI3K-mTOR inhibitor BEZ235 demonstrated the highest synergistic cytotoxic effect in combination with CsA. Given the prominent role of the PI3K/Akt/mTOR signaling pathway in T-ALL pathogenesis, this drug combination was pursued further. Enhanced cytoxic effect of the combination BEZ235 and CsA was confirmed in at least two other T-ALL cell lines (CCRF-CEM and MOLT-3; Figure 5D and Supplementary Figure S2) and three primary T-ALL xenografts (T-ALL#12, T-ALL#15 and T-ALL#19; Figure 5E and Supplementary Figure S2). Similar results were obtained using other Cn inhibitors such as CN585 or FK-506 (Figure 5F and 5G and Supplementary Figure S2).

### The synergistic anti-leukemic effect of combined PI3K-mTOR pathway and Cn inhibition is mainly due to inhibition of AKT

**Figure 6:**
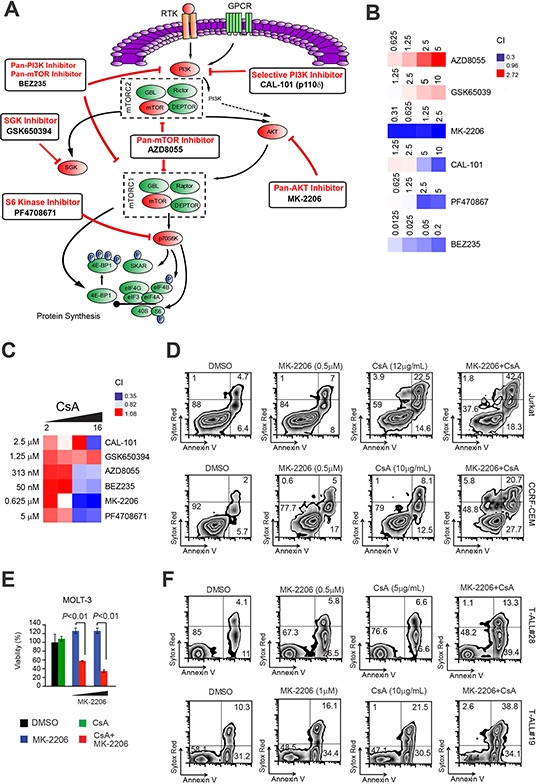
Inhibition of AKT is responsible for the synergistic anti-leukemic effect of PI3K-mTOR pathway inhibition with Cn inhibition **A.** Schematic representation of key elements of the PI3K/AKT/mTOR signaling pathway and specific inhibitors. Pathway inhibition is shown in red. **B.** Heat map representation of combination indexes (CI) of tested pathway inhibitors (used at different concentrations; shown) with a fixed concentration of the Cn inhibitor CsA (8 μg/mL) in Jurkat T-ALL cells. Concentrations are indicated in the micro-Molar (μM) range for all inhibitors. CI>1.1 indicates antagonism, CI<1 indicates synergism. **C.** Heat map representation of Combination indexes (CI) of tested pathway inhibitors (used at a fixed dose, shown) with different concentrations of the Cn inhibitor CsA (2, 4, 8, 16 μg/mL) in Jurkat T-ALL cells. **D.** (top panels) Representative plots of apoptosis in Jurkat T-ALL cells treated *in vitro* with vehicle only, MK-2206 (0.5 μM), CsA (12 μg/mL) or MK-2206 plus CsA (MK-2206 + CsA; 0.5 μM and 12 μg/mL, respectively). (**D**, bottom panels) Representative plots of apoptosis in CCRF-CEM T-ALL cells treated *in vitro* with vehicle only, MK-2206 (0.5 μM), CsA (10 μg/mL) or MK-2206 plus CsA (MK-2206 + CsA; 0.5 μM and 10 μg/mL, respectively). **E.** Cell viability quantification in MOLT-3 T-ALL cells treated *in vitro* with vehicle only, CsA (12 μg/mL), MK-2206 (0.25 or 0.5 μM), or CsA plus MK-2206 (CsA + MK-2206; 12 μg/mL and 0.25 or 0.5 μM, respectively) for 72h. Error bars represent ± SD of triplicate experiments. **F.** (top panels) Representative plots of apoptosis in T-ALL#28 cells treated *in vitro* with vehicle only, MK-2206 (0.5 μM), CsA (5 μg/mL) or CsA plus MK-2206 (MK-2206 + CsA; 0.5 μM and 5 μg/mL, respectively). (**F**, bottom panels) Representative plots of apoptosis in T-ALL#19 cells treated *in vitro* with vehicle only, MK-2206 (1 μM), CsA (10 μg/mL) or CsA plus MK-2206 (MK-2206 + CsA ; 1 μM and 10 μg/mL, respectively).

In 50-75% of T-ALL patients, PI3K/Akt/mTOR signaling pathway is constitutively active and negatively affects patient outcome [18]. To try and dissect the level at which PI3K/mTOR inhibition synergizes with Cn inhibition, we make use of selective inhibitors for key components of the pathway (Figure 6A). We thus treated Jurkat T-ALL cells with increasing doses of drugs targeting mTOR (AZD8055), PI3K (CAL-101), AKT (MK-2206), p70 S6 Kinase (PF-4708671) and SGK (GSK650394) alone or in combination with CsA. Analysis of drug interactions using the CI, disclosed that AKT inhibition by MK-2206 was the most effective in synergizing with the Cn inhibitor CsA (Figure 6B and 6C). Interestingly, the cytotoxic effect of joint AKT and Cn inhibition was superior to that obtained with BEZ235. Given these results, we proceeded in testing the novel combination MK-2206 and CsA in additional T-ALL cell lines and xenografts. To this end, T-ALL cell lines (Jurkat, CCRF-CEM and MOLT-3) were treated with vehicle, MK-2206, CsA or the combination MK-2206 plus CsA and evaluated for apoptosis induction and loss of viability after 48-72h. Consistent with our previous findings, all T-ALL cell lines tested showed an enhanced apoptotic response and loss of viability with the combination treatment compared to each single drug only (Figure 6D and 6E). Similar results were obtained when other Cn inhibitors (CN585 or FK-506) were substituted for CsA (Supplementary Figure S3). Analogously, primary T-ALL xenografts treated with the same combination treatment consisting of the AKT inhibitor MK-2206 and a Cn inhibitor (CsA/CN585/FK-506) showed an enhanced apoptotic response and loss of viability with the combination treatment compared to each single drug only (Figure 6F and Supplementary Figure S3).

We also evaluated the effects of these drugs on activated peripheral blood mononuclear cells (PBMCs; where CD3+ T cells comprised 40-70% of cell population, data not shown). PBMCs were isolated from normal donors and treated with different concentrations of the Cn inhibitors (CsA, FK-506 and CN585) or PI3K/AKT/mTOR pathway inhibitors BEZ235 and MK-2206, before being evaluated for apoptosis induction. We found that PBMCs remained highly viable even at very high concentrations (Supplementary Figure S4). In addition, combination treatments between MK-2206 and Cn inhibitors showed a very modest increase in the apoptotic effects (compared to single drug treatments) even at high drug concentrations, suggesting a favourable therapeutic index (Supplementary Figure S4).

### The anti-leukemic effect of joint inhibition of AKT and Cn is mediated through the down-regulation of multiple anti-apoptotic proteins including Mcl-1, Claspin and X-linked inhibitor of apoptosis protein (XIAP)

**Figure 7:**
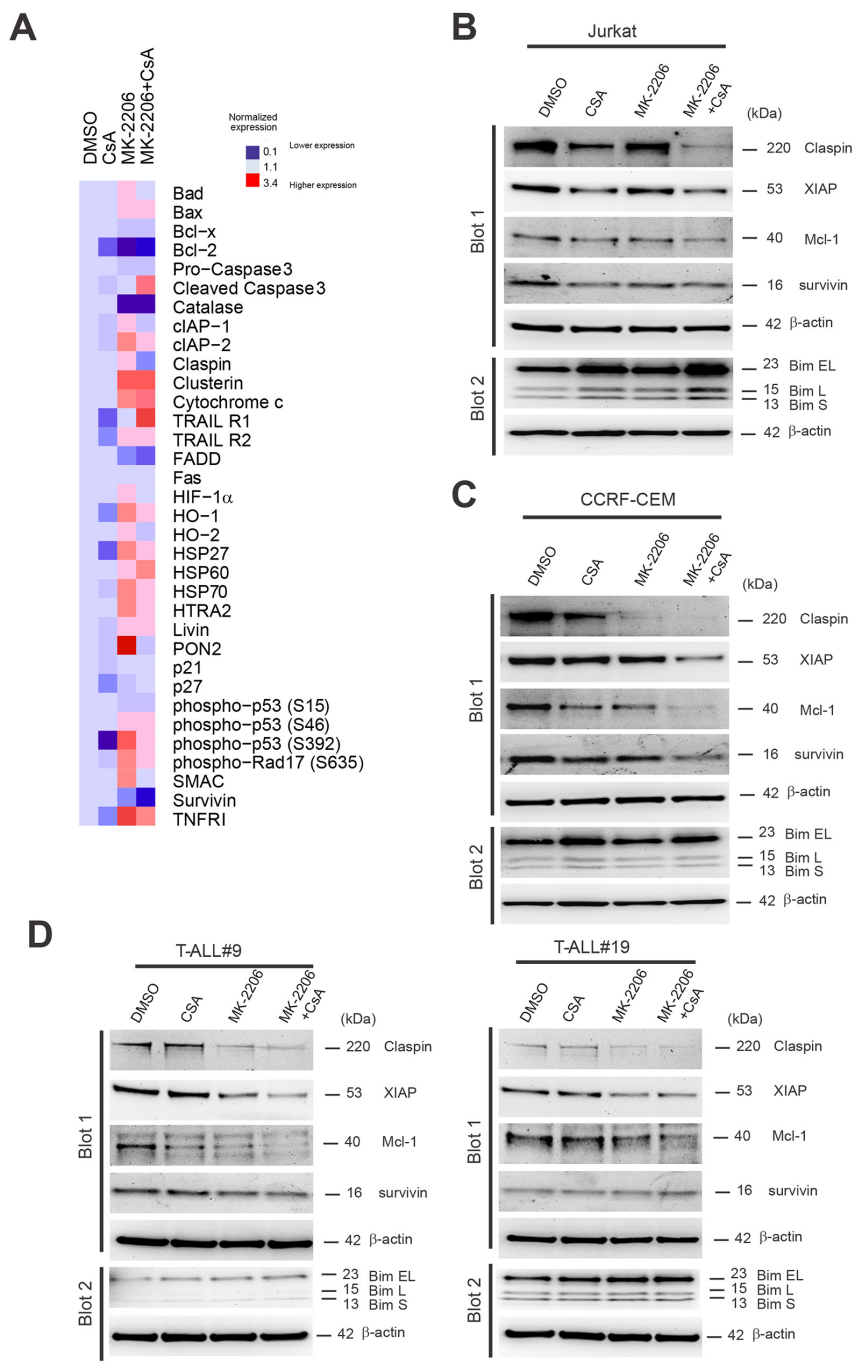
Down-regulation of multiple anti-apoptotic proteins including Mcl-1, Claspin and X-linked inhibitor of apoptosis protein (XIAP) are associated with the anti-leukemic effect of joint inhibition of AKT and Cn **A.** Whole cell extracts from Jurkat cells treated for 24h with vehicle only, MK-2206 (0.5μM), CsA (10 μg/mL) or MK-2206 plus CsA (MK-2206 + CsA; 0.5 μM and 10μg/mL, respectively) in combination were hybridized to human apoptosis arrays. Heat map representation of apoptotic protein expression relative to DMSO control is shown. **B.** Western blot analysis of Claspin, XIAP, survivin, Mcl-1 and Bim expression in Jurkat T-ALL cells treated for 24h with vehicle only, MK-2206 (0.5 μM), CsA (10 μg/mL) or MK-2206 plus CsA (MK-2206 + CsA; 0.5 μM and 10μg/mL, respectively). β-actin is shown as loading control. **C.** Western blot analysis of Claspin, XIAP, survivin, Mcl-1 and Bim expression in CCRF-CEM T-ALL cells treated for 24h with vehicle only, MK-2206 (0.5 μM), CsA (10 μg/mL) or MK-2206 plus CsA (MK + CsA; 0.5 μM and 10μg/mL, respectively). β-actin is shown as loading control. **D.** Western blot analysis of Claspin, XIAP, survivin, Mcl-1 and Bim expression in T-ALL#9 (left) and T-ALL#19 (right) xenograft cells treated for 24h with vehicle only, MK-2206 (5 μM), CsA (12 or 10 μg/mL) or MK-2206 plus CsA (MK-2206 + CsA; 5 μM and 12 or 10μg/mL, respectively). β-actin is shown as loading control.

To assess which survival factors were responsible for the strong anti-leukemic effects of the combination treatment MK-2206 and CsA, we used antibody-based apoptosis arrays. To this end, Jurkat cells were treated for 24h with vehicle, MK-2206, CsA or the combination MK-2206 plus CsA. Cell lysates were then hybridized to human apoptosis arrays, which allow the simultaneous detection of 35 apoptosis-related proteins. In Jurkat cells, the combination treatment MK-2206 and CsA determined a strong induction of cleaved Caspase 3, which was associated with a strong reduction in numerous anti-apoptotic proteins including survivin, Claspin and Bcl-2 (Figure 7A). Some of the most differentially regulated anti-apoptotic proteins in our arrays (survivin and Claspin) and three addition apoptosis related proteins either not present in the arrays (Mcl-1, Bim) or whose expression could not be reliably determined (XIAP) were investigated by IB in independent samples (Figure 7B). We found that the combination treatment MK-2206 plus CsA determined a marked decrease in the expression of Claspin, Mcl-1 and an increase in Bim isoforms in Jurkat T-ALL cells. XIAP and survivin levels were also decreased compared to vehicle control. Further analysis in two other T-ALL cell lines (CCRF-CEM and MOLT-3; Figure 7C and Supplementary Figure S5) and three primary samples (Figure 7D and Supplementary Figure S5) disclosed that Mcl-1 and Claspin were consistently down-regulated in all cases, while XIAP down-regulation was less consistent.

## DISCUSSION

Using an unbiased proteomic approach to identify proteins possibly relevant to the oncogenic properties of Cn in T-ALL, we identified novel and known PPP3CA-interacting proteins implicated in numerous cellular signaling pathways, including eIF2 signaling, cell cycle control, mTOR signaling and 14-3-3 mediated signaling. Importantly, several proteins implicated in leukemia pathobiology emerged amongst the novel PPP3CA interactors such as Rb, NPM1, BCL11b, GSK3β and KDM1 (also known as LSD1). Of these, GSK3β has been described to interact with Cn in neuron-derived cells [19] and recently reported by us to interact with PPP3CA in T-ALL cells [13]. Future studies will need to be conducted to evaluate the significance of these additional interactions.

Calcium signaling (and thus Cn-NFAT signaling) has been linked to numerous aspects of tumorigenesis, influencing motility, angiogenesis, genotoxicity, gene transcription, telomerase activity, differentiation, cell cycle progression and apoptosis [20]. Interestingly, from our proteomics analysis we identified protein synthesis and translational regulation as the most significantly enriched canonical pathways associated with our PPP3CA-binding proteins. Protein synthesis in eukaryotic cells is a complex process that requires cooperation among a large number of factors and comprises different phases (initiation, elongation and termination). Regulation of translation initiation is the main control point for mRNA translation. mTOR regulates translation initiation altering the phosphorylation of p70 S6 kinase (p70 S6K), which phosphorylates 40S ribosomal protein S6 (S6RP), and by phosphorylating the eukaryotic initiation factor 4E-binding protein 4E-BP1. Translation initiation can also be regulated through the phosphorylation of eIF2α on S51, leading to inhibition of protein synthesis [21-25]. We found that modulation of Cn activity had a mixed effect on key players involved in protein synthesis and translation regulation. In fact, optimal activation of Cn activity determined an early and transient (within 6h from stimulation) increase of phospho-p70 S6K, phospho-eIF4E and phospho-S6RP indicative of increased protein synthesis and mRNA translation, while eIF2α phosphorylation prevailed at late time points (24h from stimulation). On the other hand, inhibition of Cn activity was consistently associated with increased eIF2α phosphorylation and decreased phosphorylation of p70 S6K and S6RP.

By inhibiting pathways enriched in our PPP3CA protein complex we identified the dual PI3K-mTOR inhibitor BEZ235 as a promising drug in synergizing with Cn inhibitors to promote T-ALL cell death. Further studies to dissect the key element/s of the PI3K/AKT/mTOR pathway whose inhibition synergize best with Cn inhibitors, identified AKT as the key component to inhibit. Mechanistically, we find that joint AKT and Cn inhibition promotes cytotoxicity in T-ALL cells through the down-regulation of key anti-apoptotic proteins including Mcl-1, Claspin and XIAP. Of these, Mcl-1 down-regulation emerged as the most preserved mechanism across T-ALL cell lines and xenografts. Mcl-1 is a critical mediator of cell survival, especially of hematopoietic cells [26], and its expression is regulated through complex mechanisms at both transcriptional and post-transcriptional levels [27]. It is likely that Mcl-1 protein reduction following joint Cn and AKT inhibition is due to at least two mechanisms including phosphorylated eIF2α inhibition of *Mcl-1* mRNA translation [28, 29] and increased ubiquitin-dependent proteasomal degradation of Mcl-1 protein through augmented GSK3 dependent phosphorylation of Mcl-1 [30, 31].

The PI3K/AKT as well as mTOR pathways are two interconnected signaling pathways affecting numerous cellular functions, including metabolism, growth and survival [32, 33]. In 50-75% of T-ALL patients, this pathway is constitutively active and negatively affects patient outcome [18].

MK-2206 is a novel, orally active, allosteric AKT inhibitor, which is under development for the treatment of solid tumors. MK-2206 is a potent and selective inhibitor for AKT, whose efficacy has been proven in preclinical models of human cancer [34, 35]. In T-ALL, MK-2206 has been shown to be cytotoxic to T-ALL cell lines and to possibly target a cell subset enriched in leukemia initiating cells (LICs) in primary T-ALL samples [36]. In addition, AKT inhibition by MK-2206 has been shown to be able to reverse glucocorticoid resistance in T-ALL [37]. Thus, MK2206 has entered numerous phase I/II clinical trials for treating solid tumors and acute myeloid leukemia.

It is believed that difficulty in eradicating tumors could result from conventional treatments targeting the bulk of tumor cells, but not the putative Cancer Stem Cells (CSCs) or LICs, thus strategies aimed at eliminating these cells could have significant clinical implications. MK-2206 has been shown to be able to markedly reduce the CD34^+^/CD7^−^/CD4^−^ T-ALL subset [36], which has been reported to be enriched in LICs, in addition leukemia-initiating cell activity has been shown to require Cn in T-ALL [15]. Thus, it could be expected that joint pharmacological inhibition of PI3K/AKT/mTOR together with Cn inhibition in T-ALL could effectively deplete putative LICs contributing to improved clinical response and reduced risk of relapse.

In conclusion, our study has uncovered novel signaling pathways which interact with the Cn-NFAT signaling pathway in T-ALL cells including eIF2 signaling and p70 S6K/mTOR signaling. Interestingly, we demonstrate an enhanced anti-leukemic effect of combination strategies involving joint inhibition of AKT with Cn inhibition. Hopefully, the identification of novel interactions between canonical pathways deregulated in T-ALL for which clinically relevant inhibitors are available (such as Cn-NFAT and PI3K/AKT/mTOR pathways) could lead to to a more effective therapy of T-ALL patients still facing a poor prognosis.

## MATERIALS AND METHODS

### Affinity purification mass spectrometry and bioinformatics

For the purification of Cn-associated proteins, 12 × 10^9^ Jurkat cells stably expressing FLAG-HA tagged PPP3CA* constitutively active mutant (F/H PPP3CA*) and control Jurkat cells were harvested by centrifugation, washed in cold PBS and resuspended in CO-IP lysis buffer (50 mM Tris-HCl pH 7.9, 150 mM NaCl, 1 mM EDTA, 0.1% NP-40) supplemented with phosphatase and protease inhibitor cocktails. The lysate was cleared by centrifugation at 13.000 g for 30 min. Whole cell extracts were incubated for 16 h (at 4°C with rotation) with anti-FLAG M2 agarose beads (Sigma-Aldrich, Saint Louis, MO, USA) (1% v/v) equilibrated in BC100 (20 mM Tris-HCl pH 7.9, 100 mM NaCl, 10% glycerol, 1 mM EDTA). Beads were washed 3 times with BC100 containing 0.3% Triton X-100, and bound proteins were eluted with 4 bead volumes of BC100 containing 0.5 mg/mL of FLAG peptide (Sigma-Aldrich) for 6 h. The FLAG affinity purified complexes were further immunopurified by using 30 μL of anti-HA conjugated beads (Sigma-Aldrich). After incubation for 16 h, HA beads were washed 4 times with BC-100 containing 0.2% Triton X-100 in spin columns (Pierce, Pero, Italy) and eluted under native conditions using HA peptide (Roche, Burgess Hill, U.K.). Ten percent of the eluate was resolved on SDS-PAGE and silver stained using the silverquest kit (Invitrogen, Paisley, U.K.). The remaining material was TCA precipitated and subsequently analysed by MS at the Taplin Biological Mass Spectrometry facility (Harvard Medical School, Boston, MA). After compiling and filtering the data (proteins represented by at least three unique peptides, and proteins that were not present in the control IP), the protein list was queried on the DAVID functional annotation clustering platform to identify protein complex functions [38]. With medium stringency threshold and redundancy checking, 17 annotation clusters were finally generated to represent biological functions enriched by PPP3CA-interacting proteins. Protein interaction networks were created with Cytoscape 2.7.0 [39]. The filtered list was also run on PANTHER and STRING (Search Tool for the Retrieval of Interacting Genes/Proteins) software.

### Inhibitors and drugs

BEZ235, CAL-101, GSK650394, Roscovitine, Salubrinal, AZD8055, MK-2206 and PF4708671 were from Selleck Chemicals LLC (Houston, TX, USA). CN585 (6-(3,4-Dichlorophenyl)-4-(N,N-dimethylaminoethylthio)-2-phenyl-pyrimidine) was from Merck Millipore (Merck, Damstadt, Germany). Ionomycin, PMA, CsA, FK-506 and BV-02 were from Sigma-Aldrich.

### Transfection and immunoprecipitation

Human embryonic kidney (HEK) 293T cells were transfected with expression vectors encoding pcDNA3-F/H-PPP3CA* and each of the expression vectors encoding the novel PPP3CA interacting partners (RBBP4, hnRNPU, KHSRP, IFI-16, FLNa, RPA2, FUS, ARG1, STK38, PRDX1, DNAJA2, GSK3β, HSPA8, KIF-11, PRMT5, BCL11b and NPM1) using JetPEI transfection reagent (Polypus-transfection Inc., NY, USA). Fourty hours after transfection, cells were lysed in CO-IP buffer (50 mM Tris-HCl pH 7.9, 150 mM NaCl, 1 mM EDTA, 0.1% NP-40 and protease inhibitors). For IP, cell lysates were incubated with anti-HA or anti-FLAG M2 affinity gel beads (Sigma-Aldrich) overnight at 4°C. Beads were washed five times with lysis buffer and proteins were eluted by incubating the beads with HA peptide (1mg/ml, Roche) or FLAG peptide (1mg/ml, Sigma-Aldrich) or boiled in 5X SDS-Laemmli's sample buffer. Immune complexes were analyzed by SDS-PAGE and immunoblot. For His-tagged proteins, transfected cells were lysed in TEN buffer (20 mM Tris-HCl pH 7.5, 150 mM NaCl, 5 mM MgCl_2_, 0.1% NP-40, 10 mM Imidazole, 10% Glycerol) supplemented with protease inhibitors. Cell lysates were incubated with HisPur Cobalt resin (Pierce) overnight at 4°C. Beads were washed three times with lysis buffer and proteins were eluted by incubating the beads with 300 mM Imidazole elution buffer. Immune complexes were analyzed by SDS-PAGE and WB. For IP of endogenous PPP3CA bound proteins in T-ALL cells, we lysed 100-150×10^6^ T-ALL cells for 30 min in RIPA lysis buffer supplemented with phosphatase and protease inhibitor cocktails. After centrifugation, the cell lysates (diluted 1:3 with CO-IP buffer) were pre-cleared with TrueBlot® anti-Mouse Ig IP Beads (eBioscience, San Diego, CA, USA) before being incubated overnight with 5 μg of mouse antibody against PPP3CA (BD Pharmingen, Oxford, U.K.) or irrelevant mouse Ig (Santa Cruz Biotechnology, Heidelberg, Germany). Subsequently, samples were incubated for 2h with TrueBlot® anti-Mouse Ig IP Beads and IP washed 5 times with CO-IP buffer. Immune complexes were then analyzed by SDS-PAGE and immunoblot.

### Cell culture, cell lines and primary leukemia xenografts

HEK 293T were maintained in DMEM containing 10% fetal bovine serum (FBS) and 0.05 mg/ml penicillin/streptomycin. T-ALL cell lines were maintained in RPMI-1640 media supplemented with 10% FBS and 0.05mg/ml penicillin/streptomycin (complete medium). Primary xenograft T-ALL cells were expanded *in vivo* via i.v. injection in NOD SCID IL2Rγ^null^ (NSG) immunodeficient mice. Primary T-ALL cells from xenografted mice were cultured *in vitro* in MEM-alpha media supplemented with 10% human serum and cytokines [40] or RPMI-1640 media supplemented with 20% FBS for the duration of functional assays (48 h). Peripheral blood samples from healthy donors were collected using heparinized tubes and processed by Ficoll (GE Healthcare Life Sciences, Milan, Italy) density gradient centrifugation to isolate PBMCs. Cells were plated 1×10^6^/mL per well in complete medium and stimulated with a mixture of anti-CD3 (OKT3; 10μg/mL) and 50 ng/mL human recombinant IL-2 (Peprotech, Rocky Hill, New Jersey) to induce proliferation. Drugs were added to PBMCs for 48 h before evaluation of apoptosis induction.

### Western blotting and immunoprecipitation

Total cell lysates were prepared using RIPA lysis buffer supplemented with phosphatase inhibitor cocktail set I and II (Sigma-Aldrich) and protease inhibitor cocktail tablets (Roche) and normalized for protein concentration using the BCA method (Pierce). For Western blotting, protein samples were separated on 4-12% gradient Tris-Glycine or 3-8% Tris-Acetate SDS-PAGE (Invitrogen) and transferred to PVDF membrane (Millipore, Watford, U.K.). Antibodies against c-myc (9E10), NFATc2 and Claspin were from Santa Cruz Biotechnology; antibodies recognizing GSK3β, PPP3CA, β-actin, MCM-4, FLAG epitope, GST, phospho-mTOR (S2448), mTOR, phospho-AKT (S473), AKT, phospho-eIF2α (S51), eIF2α, phospho-eIF4E (S209), phospho-p70 S6K (T389), XIAP, Mcl-1, survivin, Claspin, Bim, phospho-S6RP (S235/236) and p70 S6K, were from Cell Signaling Technologies (Danvers, MA, USA); HA epitope antibody was from Roche; mouse anti-human Rb protein (BD Pharmingen), rabbit anti-BCL11b (Bethyl Laboratories Inc., Montomery, USA); mouse FLAG epitope antibody (M2), NFATc2 antibody (HPA 008789) were from Sigma-Aldrich; V5 tag antibody (E10/V4RR) was from Thermo Scientific (Waltham, MA, USA) and Green Fluorescent Protein antibody (A11122) was from Invitrogen.

### Human apoptosis array

We analysed the expression profiles of 35 apoptosis-related proteins on cellular extracts using the Human Apoptosis Array (R&D Systems, Minneapolis, USA). Approximately 150 μg of cell lysates were incubated overnight with the Human Apoptosis Array. The BioRad ChemiDoc XRS Imager was used to capture the signals from the arrays. The density of each spot was quantified by Quantity One software (BioRad). Raw signal intensities in each array were taken average across duplicate spots for each probe and negative control probe (PBS) was subtracted. Then processed data were expressed relative to DMSO control and heat map generated using GenePattern [41].

### Constructs and molecular cloning

FLAG tagged PPP3CA (F-PPP3CA; nt 1-1566), constitutively active FLAG-tagged PPP3CA mutant lacking the calmodulin binding and autoinhibitory domains (F-PPP3CA*; nt 1-1170), double tagged FLAG/HA PPP3CA* mutant (F/H-PPP3CA*), KIF-11, PRMT5, BCL11b and NPM1 expression vectors (in pcDNA3 backbone) were generated by standard cloning procedures. Expression constructs for RBBP4, hnRNPU, KHSRP, IFI-16, FLNa, RPA2, PKM2, FUS, ARG1, STK38, PRDX1, DNAJA2, GSK3β and HSPA8 were from Addgene.

The retroviral vector (MigR1tomato-F/H PPP3CA*), expressing the red fluorescent protein Tomato and F/H-PPP3CA* was generated by subcloning F/H-PPP3CA* from pcDNA3-F/H-PPP3CA* into the MigR1tomato vector using BglII and XhoI restriction sites.

### Retroviral constructs and viral production

Retroviral particles were generated according to standard protocols.

### Cell viability assays and flow cytometry

Cell viability in T-ALL cell lines was analyzed via the bioluminescent method Vialight plus (Lonza, Basel, Switzerland) after 72 h. This assay allows bioluminescent detection of cellular ATP as a measure of viability. Measurement of ATP is the most accurate, effective, and direct way of determining the number of living cells in culture. We also analyzed apoptosis after 48-72 h by flow cytometry (FACS) after staining with Annexin V-FITC (Roche) and SYTOX Red dead cell stain (Invitrogen). The samples were collected on a FACSCalibur (BD Biosciences, Milan, Italy) using Cell Quest software (BD Biosciences), and analysed with FlowJo (Tree Star).

### Statistical analysis

Statistical analysis was performed by Student's *t*-test and Mann-Whitney *U* test. All statistical tests were two sided and *P*<0.05 was considered statistically significant. We analyzed drug synergism using the median-effect method of Chou and Talay [17] and used the CalcuSyn software (Biosoft, Cambridge, UK) to calculate the combination index (CI). CI values below 1, equal to 1, and above 1.1 represent synergism, additivity, and antagonism, respectively.

## SUPPLEMENTARY FIGURES AND TABLES






