# Supplementary material for: Calcineurin complex isolated from T-cell acute lymphoblastic leukemia (T-ALL) cells identifies new signaling pathways including mTOR/AKT/S6K whose inhibition synergize with calcineurin inhibition to promote T-ALL cell death
Source: Oncotarget. 2016 Jun 10;7(29):45715–29. doi: 10.18632/oncotarget.9933 (PMC5216755; doi:10.18632/oncotarget.9933)
Supplement: Supplementary file 1 [file oncotarget-07-45715-s001.pdf]

Calcineurin complex isolated from T-cell acute lymphoblastic leukemia (T-ALL) cells identifies new signaling pathways including mTOR/AKT/S6K whose inhibition synergize with calcineurin inhibition to promote T-ALL cell death

SUPPLEMENTARY FIGURES AND TABLES

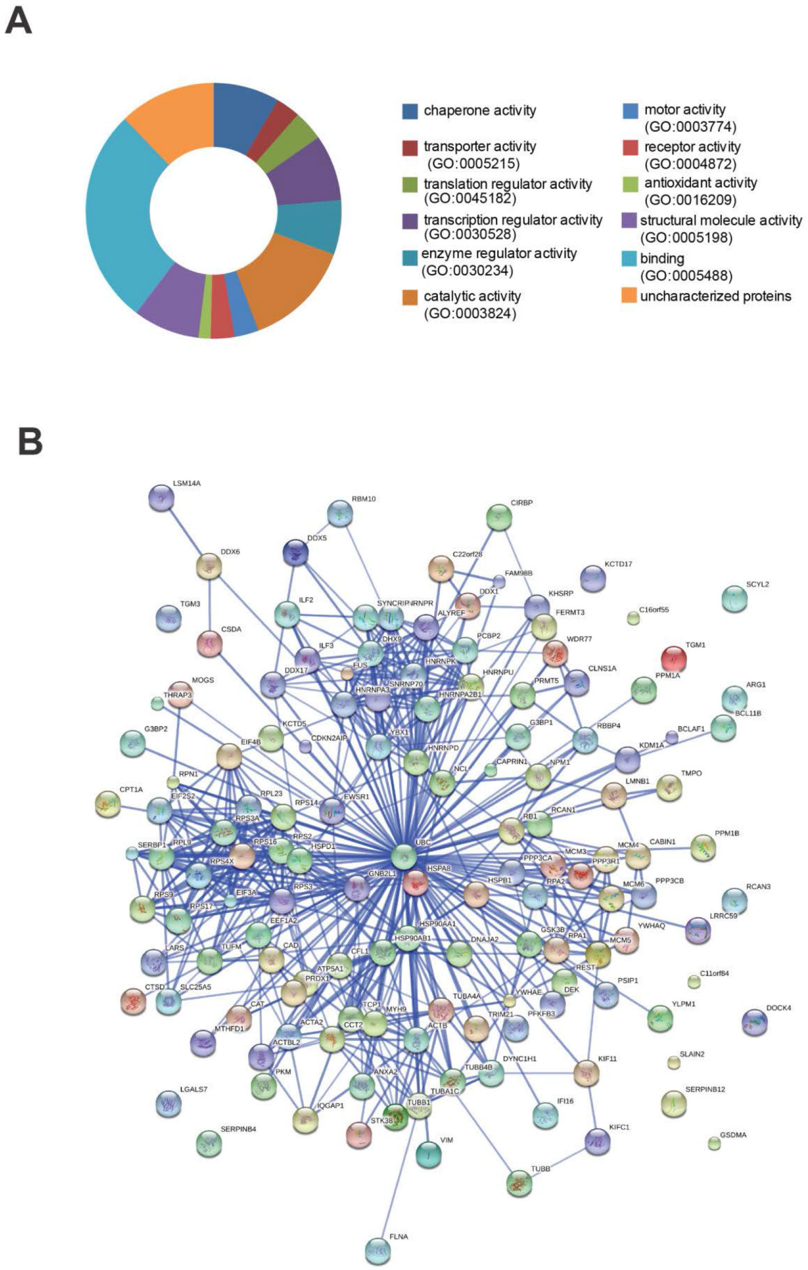

Supplementary Figure S1: Functional classification of PPP3CA-associated proteins. A. Functional classification of PPP3CA-associated proteins identified by mass spectrometry according to PANTHER software. **B.** STRING software was used to determine connections between PPP3CA-associated proteins (Confidence view) identified by mass spectrometry. Stronger associations are represented by thicker lines (high confidence associations are shown).

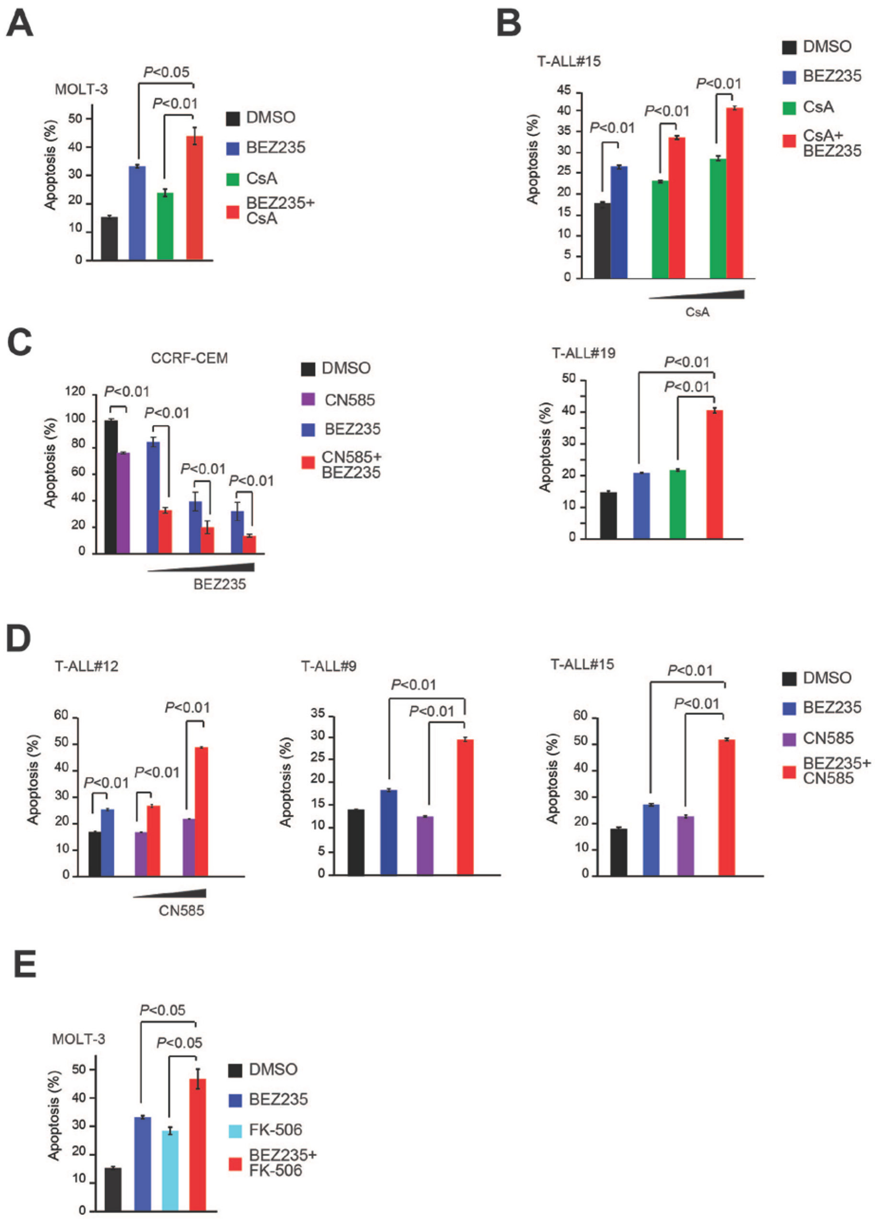

Supplementary Figure S2: Joint pharmacologic inhibition of Cn and PI3K-mTOR enhances the anti-leukemic effects of each single drug. A. Apoptosis quantification in MOLT-3 T-ALL cells treated *in vitro* with vehicle only, CsA (10 µg/mL), BEZ235 (50 nM) or BEZ235 plus CsA (BEZ235 + CsA; 50 nM and 10 µg/mL, respectively). Error bars represent ± SD of triplicate experiments. (**B.** top panel) Cell viability quantification in T-ALL#15 xenograft cells treated *in vitro* with vehicle only, CsA (10 or 15 µg/mL), BEZ235 (2 µM) or BEZ235 plus CsA (BEZ235 + CsA; 2 µM and 10 or 15 µg/mL, respectively). Error bars represent ± SD of triplicate experiments. (**B**, bottom panel) Cell viability quantification in T-ALL#19 xenograft cells treated *in vitro* with vehicle only, CsA (10 µg/mL), BEZ235 (2 µM) or BEZ235 plus CsA (BEZ235 + CsA; 2 µM and 10 µg/mL, respectively). Error bars represent ± SD of triplicate experiments. **C.** Cell viability quantification in CCRF-CEM T-ALL cells treated *in vitro* with vehicle only, BEZ235 (20-100 nM), CN585 (15 µM) or CN585 and BEZ235 (CN585 + BEZ235; 15 µM and 20-100 nM, respectively). Error bars represent ± SD of triplicate experiments. (**D.** left panel) Apoptosis quantification in T-ALL#12 cells treated *in vitro* with vehicle only, BEZ235 (2 µM), CN585 (10 or 20 µM) or BEZ235 plus CN585 (BEZ235 + CN585; 2 µM and 10 or 20 µM, respectively). Error bars represent ± SD of triplicate experiments. (**D**, middle panel) Apoptosis quantification in T-ALL#9 cells treated *in vitro* with vehicle only, BEZ235 (2 µM), CN585 (20 µM) or BEZ235 plus CN585 (BEZ235 + CN585; 2 µM and 20 µM, respectively). Error bars represent ± SD of triplicate experiments. (D, right panel) Apoptosis quantification in T-ALL#15 xenograft cells treated *in vitro* with vehicle only, CN585 (20 µM), BEZ235 (2µM) or BEZ235 plus CN585 (BEZ235 + CN585; 2µM and 20 µM, respectively). Error bars represent ± SD of triplicate experiments. **E.** Apoptosis quantification in MOLT-3 T-ALL cells treated *in vitro* with vehicle only, FK-506 (20 µM), BEZ235 (50 nM) or BEZ235 plus FK-506 (BEZ235 + FK-506; 50 nM and 20 µM, respectively). Error bars represent ± SD of triplicate experiments.

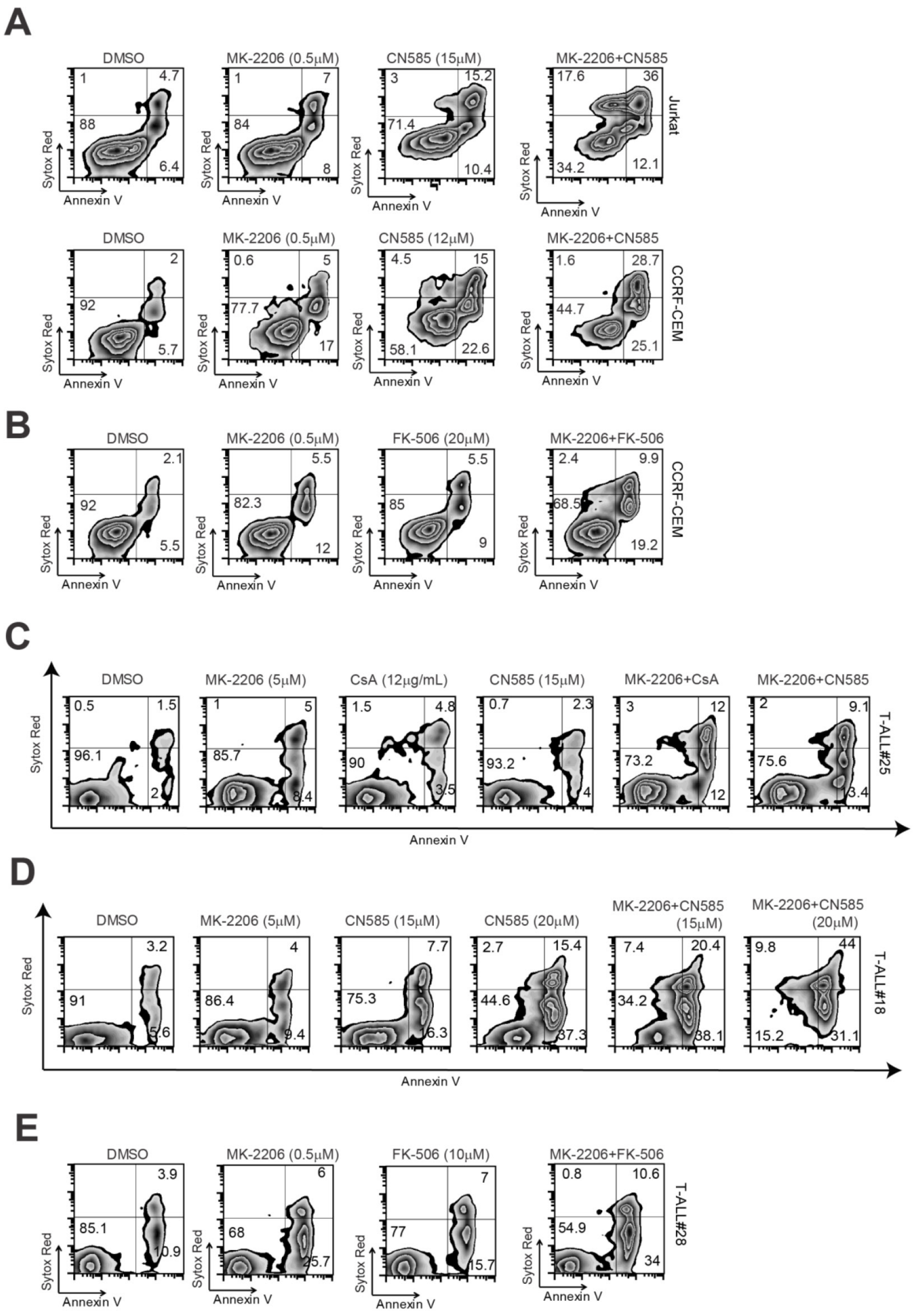

Supplementary Figure S3: Pharmacologic inhibition of Cn and AKT induces a synergistic anti-leukemic effect. (**A.** top panels) Representative plots of apoptosis in Jurkat T-ALL cells treated *in vitro* with vehicle only, MK-2206 (0.5 µM), CN585 (15 µM) or CN585 plus MK-2206 (CN585 + MK-2206; 15 µM and 0.5 µM, respectively). (**A**, bottom panels) Representative plots of apoptosis in CCRF-CEM T-ALL cells treated *in vitro* with vehicle only, MK-2206 (0.5 µM), CN585 (12 µM) or CN585 plus MK-2206 (CN585 + MK-2206; 12 µM and 0.5µM, respectively). **B.** Representative plots of apoptosis in CCRF-CEM T-ALL cells treated *in vitro* with vehicle only, MK-2206 (0.5 µM), FK-506 (20 µM) or FK-506 plus MK-2206 (FK-506 + MK-2206; 20 µM and 0.5µM, respectively). **C.** Representative plots of apoptosis in T-ALL#25 xenograft cells treated *in vitro* with vehicle only, MK-2206 (5 µM), CsA (12 µg/mL), CN585 (15 µM) or MK-2206 plus CsA or CN585 (MK-2206 + CsA; 5 µM and 12 µg/mL or MK-2206 + CN585; 5 µM and 15 µM, respectively). **D.** Representative plots of apoptosis in T-ALL#18 xenograft cells treated *in vitro* with vehicle only, MK-2206 (5 µM), CN585 (15 - 20 µM) or MK-2206 plus CN585 (MK-2206 +CN585; 5 µM and 15-20 µM, respectively). **E.** Representative plots of apoptosis in T-ALL#28 xenograft cells treated *in vitro* with vehicle only, MK-2206 (0.5 µM), FK506 (10 µM) or MK-2206 plus FK506 (MK-2206 +FK506; 0.5 µM and 10 µM, respectively).

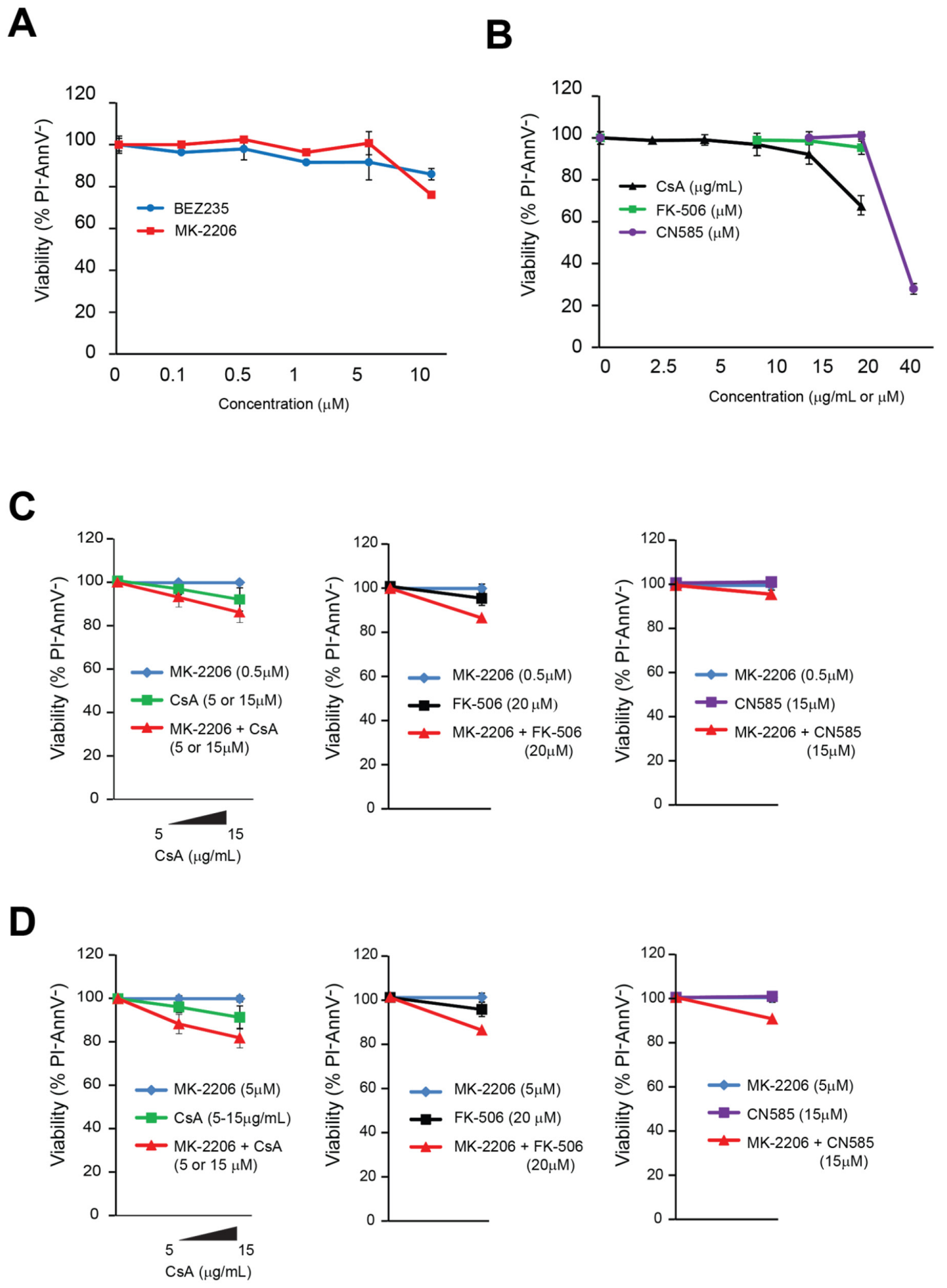

Supplementary Figure S4: Effect of pharmacologic inhibition of PI3K/mTOR or AKT or Cn on PBMCs viability. PBMCs were isolated by Ficoll density gradient, cultured in complete medium supplemented with anti-CD3 and IL-2 and exposed to different concentrations of BEZ235 and MK-2206 (panel **A.**) or to the Cn inhibitors (CsA or FK-506 or CN585; panel **B.**) for 48 hours and evaluated for viability by flow cytometry. Viability is expressed as percentage of viable cells ($AnnexinV^-PI^-$) compared to DMSO treated controls. **C.** PBMCs were cultured in complete medium supplemented with anti-CD3 and IL-2 and exposed to the AKT inhibitor MK-2206 (0.5 µM) in combination with different concentrations of the Cn inhibitor (CsA 5 or 15 µg/mL; left panel), FK-506 (20 µM; middle panel) or CN585 (15µM; right panel) for 48 hours and evaluated for viability by flow cytometry. Viability is expressed as percentage of viable cells ($AnnexinV^-PI^-$) compared to DMSO treated controls. **D.** PBMCs were cultured in complete medium supplemented with anti-CD3 and IL-2 and exposed to the AKT inhibitor MK-2206 (5 µM) in combination with different concentrations of the Cn inhibitor (CsA 5 or 15 µg/mL; left panel), FK506 (20 µM; middle panel) or CN585 (15 µM; right panel) for 48 hours and evaluated for viability by flow cytometry. Viability is expressed as percentage of viable cells ($AnnexinV^-PI^-$) compared to DMSO treated controls. For these experiments 2-4 different donors were used.

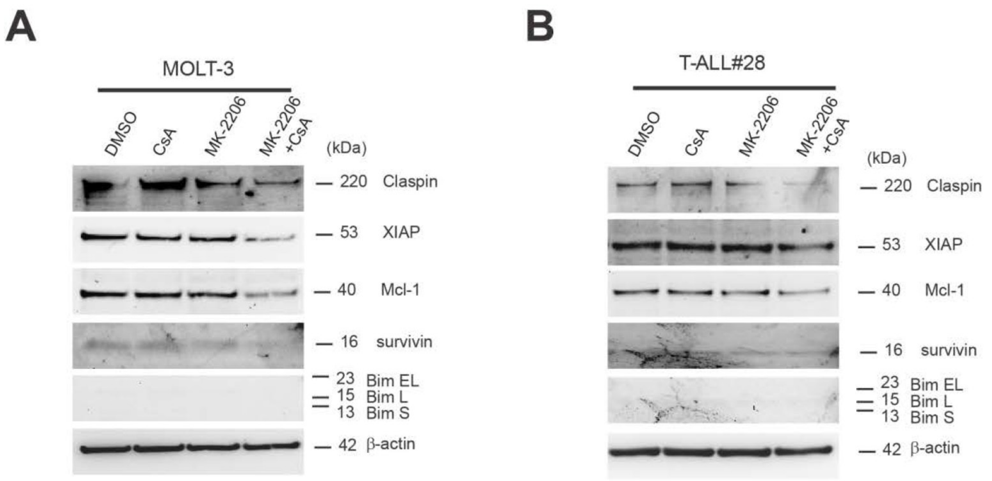

Supplementary Figure S5: Down-regulation of Mcl-1, Claspin and X-linked inhibitor of apoptosis protein (XIAP) are associated with the anti-leukemic effect of joint inhibition of AKT and Cn. A. Western blot analysis of Claspin, XIAP, survivin, Mcl-1 and Bim expression in MOLT-3 T-ALL cells treated for 24 h with vehicle only, MK-2206 (0.5 μM), CsA (12 μg/mL) or MK-2206 plus CsA (MK-2206 + CsA; 0.5 μM and 12μg/mL, respectively). β-actin is shown as loading control. **B.** Western blot analysis of Claspin, XIAP, survivin, Mcl-1 and Bim expression in T-ALL#28 xenograft cells treated for 24 h with vehicle only, MK-2206 (0.5 μM), CsA (5 μg/mL) or MK-2206 plus CsA (MK-2206 + CsA; 0.5 μM and 5 μg/mL, respectively). β-actin is shown as loading control.

Supplementary Table S1: List of PPP3CA-interacting proteins identified by mass spectrometry analysis.

See Supplementary File 1

Supplementary Table S2: List of canonical signaling pathways enriched in identified PPP3CA-interacting proteins.

See Supplementary File 2
