# Supplementary material for: Calcineurin complex isolated from T-cell acute lymphoblastic leukemia (T-ALL) cells identifies new signaling pathways including mTOR/AKT/S6K whose inhibition synergize with calcineurin inhibition to promote T-ALL cell death
Source: Oncotarget. 2016 Jun 10;7(29):45715–29. doi: 10.18632/oncotarget.9933 (PMC5216755; doi:10.18632/oncotarget.9933)
Supplement: Supplementary file 2 [file oncotarget-07-45715-s002.docx]

**Table S1.** **PPP3CA-interacting proteins identified by mass spectrometry analysis**

|  | | | | | | |  |  |
| --- | --- | --- | --- | --- | --- | --- | --- | --- |
| Interacting  Partner | knownAs  PartnerFrom  Literature | functionCluster  .representative | Number.of  unique  peptides | Total  peptides | IPI Id | geneSymbol.new | Entrez Id | Ensemble Id |
| **KIF11*** | NO | Cytoskeletal remodeling | 62 | 115 | IPI00305289.2 | KIF11 | 3832 | ENSG00000138160 |
| SCYL2 | NO | ATP binding | 49 | 80 | IPI00396218.2 | SCYL2 | 55681 | ENSG00000136021 |
| ATP5A1 | NO | Nucleotide binding | 24 | 36 | IPI00440493.2 | ATP5A1 | 498 | ENSG00000152234 |
| TUBB2C | NO | Cytoskeletal remodeling | 17 | 28 | IPI00007752.1 | TUBB4B | 10383 | ENSG00000188229 |
| PPP3R1 | NO | Signaling crosstalk | 15 | 33 | IPI00027464.8 | PPP3R1 | 5534 | ENSG00000221823 |
| MCM3 | NO | DNA replication | 15 | 17 | IPI00013214.2 | MCM3 | 4172 | ENSG00000112118 |
| PPM1B | NO | Signaling crosstalk | 14 | 20 | IPI00026612.1 | PPM1B | 5495 | ENSG00000138032 |
| RPS3 | NO | Transcriptional repression | 13 | 14 | IPI00011253.3 | RPS3 | 6188 | ENSG00000149273 |
| HNRNPK | NO | RNA splicing | 13 | 17 | IPI00216049.1 | HNRNPK | 3190 | ENSG00000165119 |
| **FLNA*** | NO | Regulation of Trafficking | 13 | 15 | IPI00302592.2 | FLNA | 2316 | ENSG00000196924 |
| G3BP1 | NO | Transcriptional regulation | 12 | 15 | IPI00012442.1 | G3BP1 | 10146 | ENSG00000145907 |
| **GSK3B*** | NO | Signaling crosstalk | 12 | 20 | IPI00028570.2 | GSK3B | 2932 | ENSG00000082701 |
| TRIM21 | NO | Chaperone and cytoskeletal regulation | 12 | 16 | IPI00018971.8 | TRIM21 | 6737 | ENSG00000132109 |
| CAPRIN1 | NO | Transcriptional repression | 11 | 13 | IPI00783872.2 | CAPRIN1 | 4076 | ENSG00000135387 |
| DDX6 | NO | Nucleotide binding | 11 | 12 | IPI00030320.4 | DDX6 | 1656 | ENSG00000110367 |
| **HNRNPU*** | NO | Nucleotide binding | 11 | 13 | IPI00479217.1 | HNRNPU | 3192 | ENSG00000153187 |
| TUBA4A | NO | Cytoskeletal remodeling | 11 | 20 | IPI00007750.1 | TUBA4A | 7277 | ENSG00000127824 |
| **STK38*** | NO | Nucleotide binding | 10 | 10 | IPI00027251.1 | STK38 | 11329 | ENSG00000112079 |
| HSPD1 | NO | Apoptosis signaling | 10 | 11 | IPI00784154.1 | HSPD1 | 3329 | ENSG00000144381 |
| EWSR1 | NO | Transcriptional regulation | 9 | 13 | IPI00009841.5 | EWSR1 | 2130 | ENSG00000182944 |
| C22orf28 | NO | Unknown | 8 | 9 | IPI00550689.3 | C22orf28 | 51493 | ENSG00000100220 |
| SERPINB4 | NO | Unknown | 8 | 9 | IPI00010303.1 | SERPINB4 | 6318 | ENSG00000206073 |
| **PRMT5*** | NO | Transcriptional regulation | 8 | 8 | IPI00064328.3 | PRMT5 | 10419 | ENSG00000100462 |
| SERBP1 | NO | RNA splicing | 8 | 10 | IPI00410693.3 | SERBP1 | 26135 | ENSG00000142864 |
| DDX1 | NO | Nucleotide binding | 8 | 8 | IPI00293655.3 | DDX1 | 1653 | ENSG00000079785 |
| HSP90AB1 | NO | Metabolic regulation | 8 | 8 | IPI00414676.6 | HSP90AB1 | 3326 | ENSG00000096384 |
| RCAN1 | YES | Differentiation regulation | 8 | 16 | IPI00023978.4 | RCAN1 | 1827 | ENSG00000159200 |
| ***PKM2*** | NO | ATP binding | 8 | 10 | IPI00220644.8 | PKM | 5315 | ENSG00000067225 |
| YLPM1 | NO | Transcriptional regulation | 7 | 8 | IPI00165434.4 | YLPM1 | 56252 | ENSG00000119596 |
| RBM10 | NO | RNA splicing | 7 | 8 | IPI00375731.1 | RBM10 | 8241 | ENSG00000182872 |
| EIF4B | NO | Protein biosynthesis | 7 | 9 | IPI00012079.3 | EIF4B | 1975 | ENSG00000063046 |
| TMPO | NO | DNA replication | 7 | 8 | IPI00030131.3 | TMPO | 7112 | ENSG00000120802 |
| NCL | NO | Cytoskeletal remodeling | 7 | 7 | IPI00444262.3\|IPI00183526.6 | NCL | 4691 | ENSG00000115053 |
| ACTB | NO | Cytoskeletal remodeling | 7 | 12 | IPI00021439.1 | ACTB | 60 | ENSG00000075624 |
| TUBB | YES | Cytoskeletal remodeling | 7 | 15 | IPI00011654.2 | TUBB | 203068 | ENSG00000196230 |
| **NPM1*** | NO | Apoptosis signaling | 7 | 9 | IPI00220740.1 | NPM1 | 4869 | ENSG00000181163 |
| **PRDX1*** | NO | Apoptosis signaling | 7 | 9 | IPI00000874.1 | PRDX1 | 5052 | ENSG00000117450 |
| ILF3 | NO | Transcriptional regulation | 6 | 6 | IPI00219330.2 | ILF3 | 3609 | ENSG00000129351 |
| HSP90AA1 | NO | Trafficking | 6 | 7 | IPI00382470.3 | HSP90AA1 | 3320 | ENSG00000080824 |
| PPP3CB | YES | Signaling crosstalk | 6 | 15 | IPI00027809.2 | PPP3CB | 5532 | ENSG00000107758 |
| CDKN2AIP | NO | RNA-binding | 6 | 6 | IPI00020991.2 | CDKN2AIP | 55602 | ENSG00000168564 |
| RCAN3 | YES | RNA-binding | 6 | 16 | IPI00007419.1 | RCAN3 | 11123 | ENSG00000117602 |
| DDX5 | NO | Nucleotide binding | 6 | 6 | IPI00017617.1 | DDX5 | 1655 | ENSG00000108654 |
| DHX9 | NO | Nucleotide binding | 6 | 6 | IPI00844578.1 | DHX9 | 1660 | ENSG00000135829 |
| YBX1 | NO | Metabolic regulation | 6 | 7 | IPI00031812.3 | YBX1 | 4904 | ENSG00000065978 |
| REST | NO | Metabolic regulation | 6 | 6 | IPI00159969.3 | REST | 5978 | ENSG00000084093 |
| ***RPA2*** | NO | DNA replication | 6 | 6 | IPI00013939.3 | RPA2 | 6118 | ENSG00000117748 |
| RPA1 | NO | DNA replication | 6 | 6 | IPI00020127.1 | RPA1 | 6117 | ENSG00000132383 |

| Interacting  Partner | knownAs  PartnerFrom  Literature | functionCluster  .representative | Number.of.  Unique  peptides | Total  peptides | IPI Id | geneSymbol.  new | Entrez Id | Ensemble Id |
| --- | --- | --- | --- | --- | --- | --- | --- | --- |
| MCM5 | NO | DNA replication | 6 | 6 | IPI00018350.3 | MCM5 | 4174 | ENSG00000100297 |
| TGM1 | NO | Cytoskeletal remodeling | 6 | 6 | IPI00305622.4 | TGM1 | 7051 | ENSG00000092295 |
| ACTBL2 | NO | Cytoskeletal remodeling | 6 | 7 | IPI00003269.1 | ACTBL2 | 345651 | ENSG00000169067 |
| TGM3 | NO | Chaperone and cytoskeletal regulation | 6 | 6 | IPI00300376.4 | TGM3 | 7053 | ENSG00000125780 |
| C11orf84 | NO | Unknown | 5 | 7 | IPI00106955.3 | C11orf84 | 144097 | ENSG00000168005 |
| CPT1A | NO | Unknown | 5 | 5 | IPI00032038.4 | CPT1A | 1374 | ENSG00000110090 |
| FAM98B | NO | Unknown | 5 | 5 | IPI00167572.4 | FAM98B | 283742 | ENSG00000171262 |
| ILF2 | NO | Transcriptional regulation | 5 | 7 | IPI00005198.2 | ILF2 | 3608 | ENSG00000143621 |
| PSIP1 | NO | Transcriptional regulation | 5 | 6 | IPI00028122.1 | PSIP1 | 11168 | ENSG00000164985 |
| RB1 | YES | Transcriptional regulation | 5 | 6 | IPI00302829.5 | RB1 | 5925 | ENSG00000139687 |
| **HSPA8*** | NO | Trafficking | 5 | 5 | IPI00003865.1 | HSPA8 | 3312 | ENSG00000109971 |
| PPP3CA | YES | Signaling crosstalk | 5 | 12 | IPI00179415.4 | PPP3CA | 5530 | ENSG00000138814 |
| HNRNPA3 | NO | RNA splicing | 5 | 5 | IPI00419373.1 | HNRNPA3 | 220988 | ENSG00000170144 |
| **FUS*** | NO | RNA splicing | 5 | 7 | IPI00221354.1 | FUS | 2521 | ENSG00000089280 |
| PCBP2 | NO | RNA splicing | 5 | 6 | IPI00012066.2 | PCBP2 | 5094 | ENSG00000197111 |
| EIF2S2 | NO | Protein biosynthesis | 5 | 5 | IPI00021728.3 | EIF2S2 | 8894 | ENSG00000125977 |
| RPL9 | NO | Protein biosynthesis | 5 | 8 | IPI00031691.1 | RPL9 | 6133 | ENSG00000163682 |
| RPS4X | NO | Protein biosynthesis | 5 | 5 | IPI00217030.1 | RPS4X | 6191 | ENSG00000198034 |
| LSM14A | NO | Protein biosynthesis | 5 | 8 | IPI00410590.5 | LSM14A | 26065 | ENSG00000257103 |
| DDX17 | NO | Nucleotide binding | 5 | 5 | IPI00023785.7 | DDX17 | 10521 | ENSG00000100201 |
| **KHSRP*** | NO | Nucleic acid transport | 5 | 5 | IPI00479786.5 | KHSRP | 8570 | ENSG00000088247 |
| HNRNPA2B1 | NO | Nucleic acid transport | 5 | 6 | IPI00386854.6 | HNRNPA2B1 | 3181 | ENSG00000122566 |
| THOC4 | NO | Nucleic acid transport | 5 | 6 | IPI00328840.9 | ALYREF | 10189 | ENSG00000183684 |
| RPS16 | NO | Chaperone and cytoskeletal regulation | 5 | 6 | IPI00221092.8 | RPS16 | 6217 | ENSG00000105193 |
| ANXA2P2 | NO | Unknown | 4 | 5 | IPI00334627.3 | ANXA2P2 | 304 | NULL |
| ARG1 | NO | Unknown | 4 | 4 | IPI00291560.4 | ARG1 | 383 | ENSG00000118520 |
| C16orf55 | NO | Unknown | 4 | 5 | IPI00043271.1 | C16orf55 | 124045 | ENSG00000167523 |
| CABIN1 | YES | Unknown | 4 | 4 | IPI00002355.1 | CABIN1 | 23523 | ENSG00000099991 |
| GNB2L1 | YES | Unknown | 4 | 4 | IPI00641950.4 | GNB2L1 | 10399 | ENSG00000204628 |
| KCTD17 | NO | Unknown | 4 | 4 | IPI00297162.5 | KCTD17 | 79734 | ENSG00000100379 |
| LGALS7 | NO | Unknown | 4 | 5 | IPI00219221.3 | LGALS7 | 3963 | ENSG00000205076 |
| SLC25A5 | NO | Unknown | 4 | 5 | IPI00007188.5 | SLC25A5 | 292 | ENSG00000005022 |
| RPS2 | NO | Transcriptional repression | 4 | 4 | IPI00013485.3 | RPS2 | 6187 | ENSG00000140988 |
| CSDA | NO | Transcriptional repression | 4 | 5 | IPI00031801.4 | CSDA | 8531 | ENSG00000060138 |
| THRAP3 | NO | Transcriptional repression | 4 | 4 | IPI00104050.3 | THRAP3 | 9967 | ENSG00000054118 |
| CIRBP | NO | Transcriptional regulation | 4 | 7 | IPI00180954.4 | CIRBP | 1153 | ENSG00000099622 |
| HSP90AA2 | NO | Trafficking | 4 | 4 | IPI00031523.4 | HSP90AA2 | 3324 | NULL |
| HSPB1 | NO | Signaling crosstalk | 4 | 4 | IPI00025512.2 | HSPB1 | 3315 | ENSG00000106211 |
| SYNCRIP | NO | RNA splicing | 4 | 4 | IPI00018140.3 | SYNCRIP | 10492 | ENSG00000135316 |
| SNRNP70 | NO | RNA splicing | 4 | 5 | IPI00219483.1 | SNRNP70 | 6625 | ENSG00000104852 |
| G3BP2 | NO | Regulation of Trafficking | 4 | 5 | IPI00009057.2 | G3BP2 | 9908 | ENSG00000138757 |
| TCP1 | NO | Protein biosynthesis | 4 | 4 | IPI00290566.1 | TCP1 | 6950 | ENSG00000120438 |
| LMNB1 | NO | Protein biosynthesis | 4 | 4 | IPI00217975.4 | LMNB1 | 4001 | ENSG00000113368 |
| EEF1A2 | NO | Protein biosynthesis | 4 | 4 | IPI00014424.1 | EEF1A2 | 1917 | ENSG00000101210 |
| RPS14 | NO | Metabolic regulation | 4 | 4 | IPI00026271.5 | RPS14 | 6208 | ENSG00000164587 |
| HNRNPD | NO | DNA replication | 4 | 6 | IPI00028888.1 | HNRNPD | 3184 | ENSG00000138668 |
| **RBBP4*** | NO | DNA replication | 4 | 4 | IPI00328319.8 | RBBP4 | 5928 | ENSG00000162521 |
| MCM6 | NO | DNA replication | 4 | 4 | IPI00031517.1 | MCM6 | 4175 | ENSG00000076003 |
| MYH9 | NO | Differentiation regulation | 4 | 4 | IPI00019502.3 | MYH9 | 4627 | ENSG00000100345 |
| RPS17 | NO | Chaperone and cytoskeletal regulation | 4 | 4 | IPI00221093.7 | RPS17 | 6218 | ENSG00000184779 |

|  |  |  |  |  |  |  |  |  |
| --- | --- | --- | --- | --- | --- | --- | --- | --- |
| Interacting  Partner | knownAs  PartnerFrom  Literature | functionCluster  .representative | Number.of.  Unique  peptides | Total  peptides | IPIId | geneSymbol  .new | Entrez  Id | Ensemble Id |
| TUBA1C | NO | Chaperone and cytoskeletal regulation | 4 | 9 | IPI00166768.3 | TUBA1C | 84790 | ENSG00000167553 |
| GSDMA | NO | Apoptosis signaling | 4 | 4 | IPI00166200.4 | GSDMA | 284110 | ENSG00000167914 |
| CTSD | NO | Unknown | 3 | 3 | IPI00011229.1 | CTSD | 1509 | ENSG00000117984 |
| KCTD5 | NO | Unknown | 3 | 4 | IPI00004506.3 | KCTD5 | 54442 | ENSG00000167977 |
| LRRC59 | NO | Unknown | 3 | 3 | IPI00396321.1 | LRRC59 | 55379 | ENSG00000108829 |
| MOGS | NO | Unknown | 3 | 3 | IPI00328170.9 | MOGS | 7841 | ENSG00000115275 |
| RPN1 | NO | Unknown | 3 | 3 | IPI00025874.2 | RPN1 | 6184 | ENSG00000163902 |
| SLAIN2 | NO | Unknown | 3 | 3 | IPI00853278.1 | SLAIN2 | 57606 | ENSG00000109171 |
| WDR77 | NO | Transcriptional repression | 3 | 3 | IPI00012202.1 | WDR77 | 79084 | ENSG00000116455 |
| KDM1A | NO | Transcriptional repression | 3 | 3 | IPI00217540.7 | KDM1 | 23028 | ENSG00000004487 |
| CAT | NO | Transcriptional repression | 3 | 3 | IPI00465436.4 | CAT | 847 | ENSG00000121691 |
| DEK | NO | Transcriptional regulation | 3 | 3 | IPI00020021.3 | DEK | 7913 | ENSG00000124795 |
| BCLAF1 | NO | Transcriptional regulation | 3 | 3 | IPI00006079.1 | BCLAF1 | 9774 | ENSG00000029363 |
| **IFI16*** | NO | Transcriptional regulation | 3 | 3 | IPI00003443.3 | IFI16 | 3428 | ENSG00000163565 |
| **BCL11B*** | NO | Transcriptional regulation | 3 | 3 | IPI00009213.1 | BCL11B | 64919 | ENSG00000127152 |
| RPL23 | NO | Trafficking | 3 | 3 | IPI00010153.5 | RPL23 | 9349 | ENSG00000125691 |
| CFL1 | NO | Trafficking | 3 | 3 | IPI00012011.6 | CFL1 | 1072 | ENSG00000172757 |
| PPM1A | NO | Signaling crosstalk | 3 | 4 | IPI00020950.3 | PPM1A | 5494 | ENSG00000100614 |
| IQGAP1 | YES | Signaling crosstalk | 3 | 3 | IPI00009342.1 | IQGAP1 | 8826 | ENSG00000140575 |
| YWHAE | NO | Signaling crosstalk | 3 | 3 | IPI00000816.1 | YWHAE | 7531 | ENSG00000108953 |
| YWHAQ | NO | Signaling crosstalk | 3 | 4 | IPI00018146.1 | YWHAQ | 10971 | ENSG00000134308 |
| PFKFB3 | NO | Signaling crosstalk | 3 | 3 | IPI00004511.3 | PFKFB3 | 5209 | ENSG00000170525 |
| HNRNPR | NO | RNA splicing | 3 | 3 | IPI00012074.3 | HNRNPR | 10236 | ENSG00000125944 |
| UBC | NO | Protein biosynthesis | 3 | 3 | IPI00179330.6 | UBC | 7316 | ENSG00000150991 |
| RPS9 | NO | Protein biosynthesis | 3 | 3 | IPI00221088.5 | RPS9 | 6203 | ENSG00000170889 |
| EIF3A | NO | Protein biosynthesis | 3 | 3 | IPI00029012.1 | EIF3A | 8661 | ENSG00000107581 |
| CCT2 | NO | Protein biosynthesis | 3 | 3 | IPI00297779.7 | CCT2 | 10576 | ENSG00000166226 |
| LARS | NO | Protein biosynthesis | 3 | 3 | IPI00103994.4 | LARS | 51520 | ENSG00000133706 |
| **DNAJA2*** | NO | Protein biosynthesis | 3 | 3 | IPI00032406.1 | DNAJA2 | 10294 | ENSG00000069345 |
| TUFM | NO | Nucleotide binding | 3 | 3 | IPI00027107.5 | TUFM | 7284 | ENSG00000178952 |
| KIFC1 | NO | Nucleotide binding | 3 | 3 | IPI00306400.9 | KIFC1 | 3833 | ENSG00000237649 |
| MTHFD1 | NO | Nucleotide binding | 3 | 3 | IPI00218342.1 | MTHFD1 | 4522 | ENSG00000100714 |
| SERPINB12 | NO | Metabolic regulation | 3 | 3 | IPI00033583.3 | SERPINB12 | 89777 | ENSG00000166634 |
| MCM4 | NO | DNA replication | 3 | 3 | IPI00018349.5 | MCM4 | 4173 | ENSG00000104738 |
| FERMT3 | NO | Cytoskeletal remodeling | 3 | 3 | IPI00216699.1 | FERMT3 | 83706 | ENSG00000149781 |
| VIM | NO | Cytoskeletal remodeling | 3 | 3 | IPI00418471.6 | VIM | 7431 | ENSG00000026025 |
| DYNC1H1 | NO | Cytoskeletal remodeling | 3 | 3 | IPI00456969.1 | DYNC1H1 | 1778 | ENSG00000197102 |
| DOCK4 | NO | Cytoskeletal remodeling | 3 | 3 | IPI00006024.4 | DOCK4 | 9732 | ENSG00000128512 |
| CLNS1A | NO | Chaperone and cytoskeletal regulation | 3 | 3 | IPI00004795.1 | CLNS1A | 1207 | ENSG00000074201 |
| RPS3A | NO | Apoptosis signaling | 3 | 4 | IPI00419880.6 | RPS3A | 6189 | ENSG00000145425 |
| CAD | NO | ATP binding | 3 | 3 | IPI00301263.2 | CAD | 790 | ENSG00000084774 |
|  |  |  |  |  |  |  |  |  |
| ***Validated interacting proteins** | |  |  |  |  |  |  |  |
| ***Not validated*** |  |  |  |  |  |  |  |  |
