# Supplementary figures and images for: Calcineurin complex isolated from T-cell acute lymphoblastic leukemia (T-ALL) cells identifies new signaling pathways including mTOR/AKT/S6K whose inhibition synergize with calcineurin inhibition to promote T-ALL cell death
Source: Oncotarget. 2016 Jun 10;7(29):45715–29. doi: 10.18632/oncotarget.9933 (PMC5216755; doi:10.18632/oncotarget.9933)

**Table S2.** **Canonical signaling pathways enriched in identified PPP3CA-interacting proteins.**

**
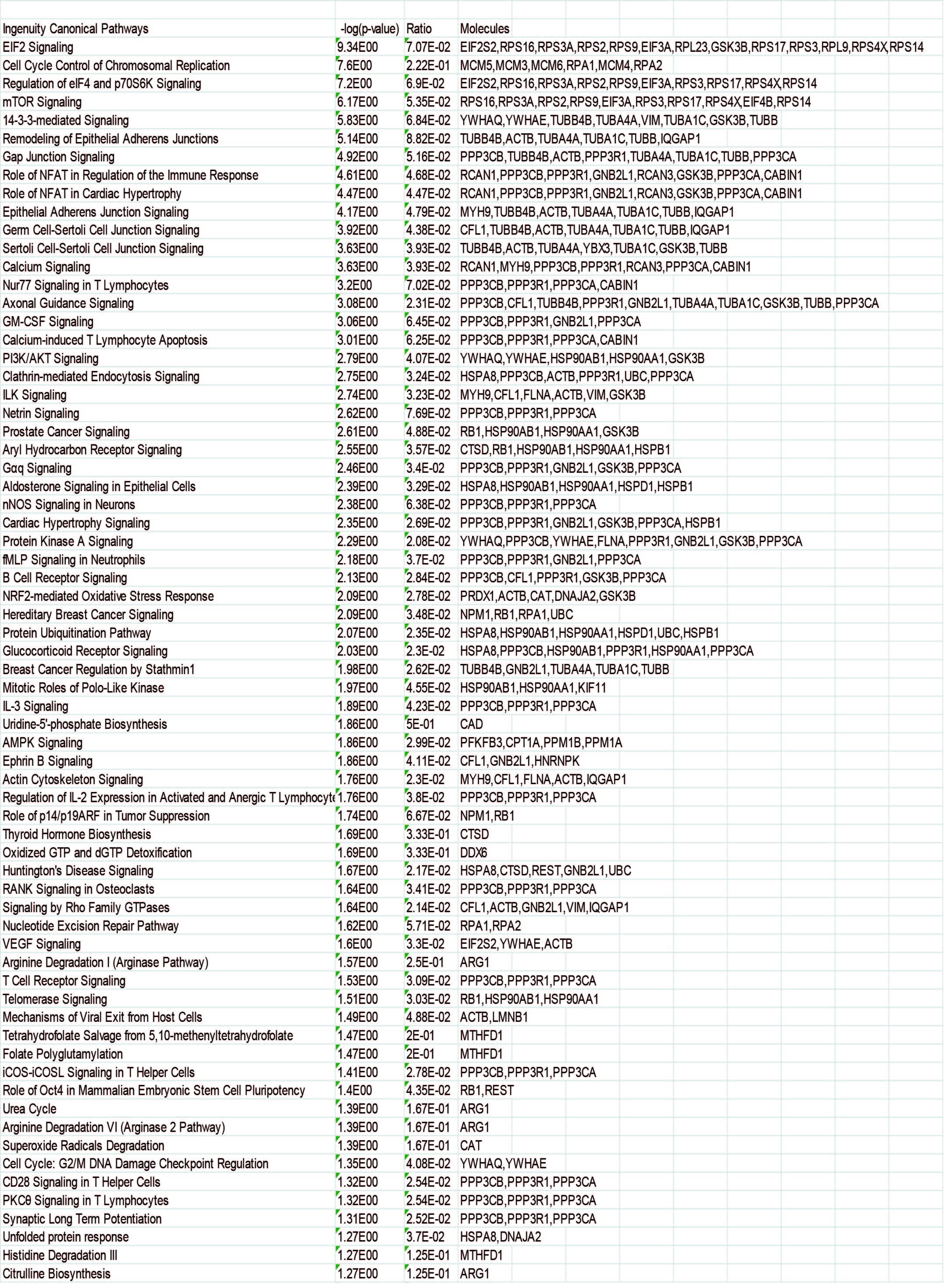
**

**
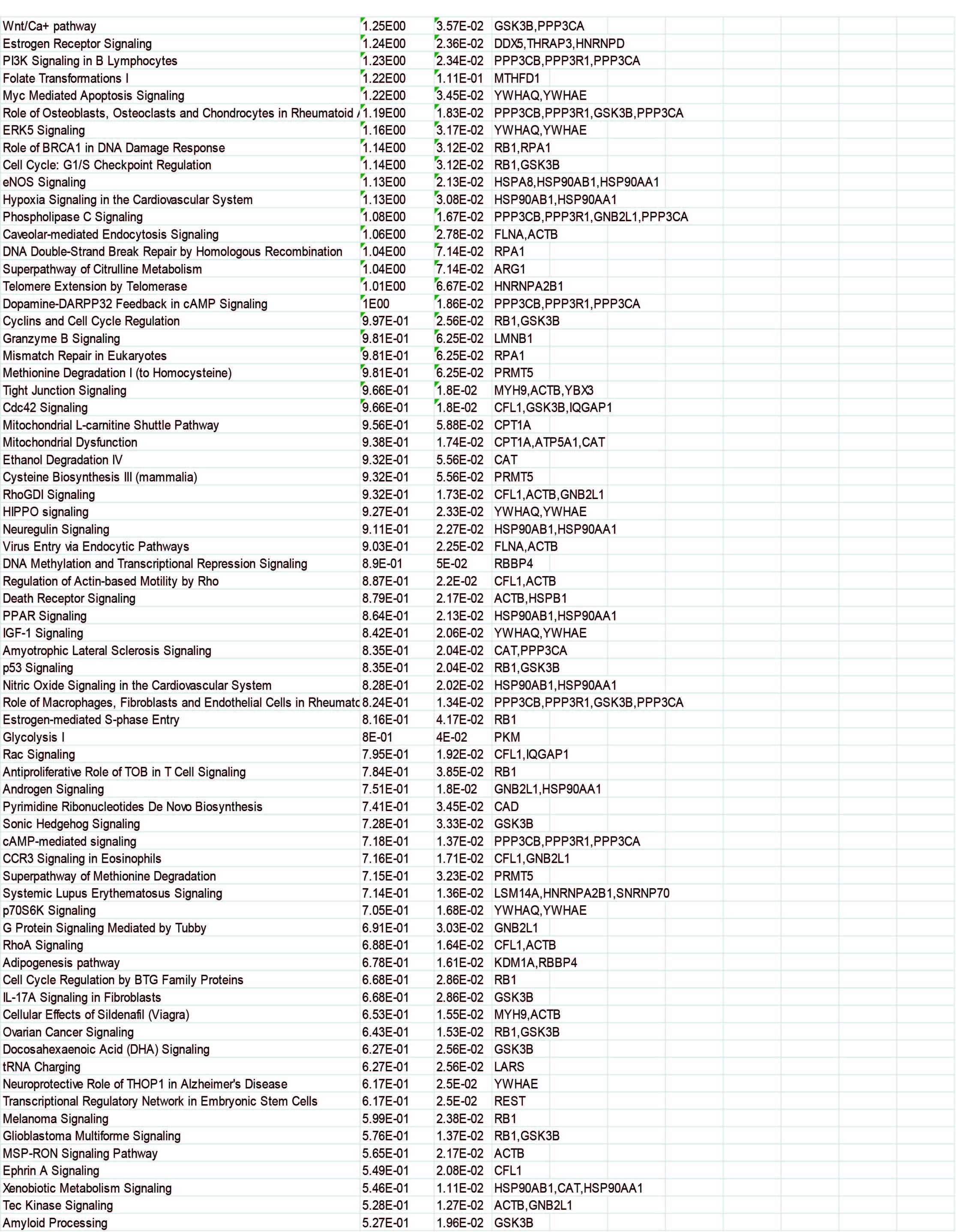
**

**
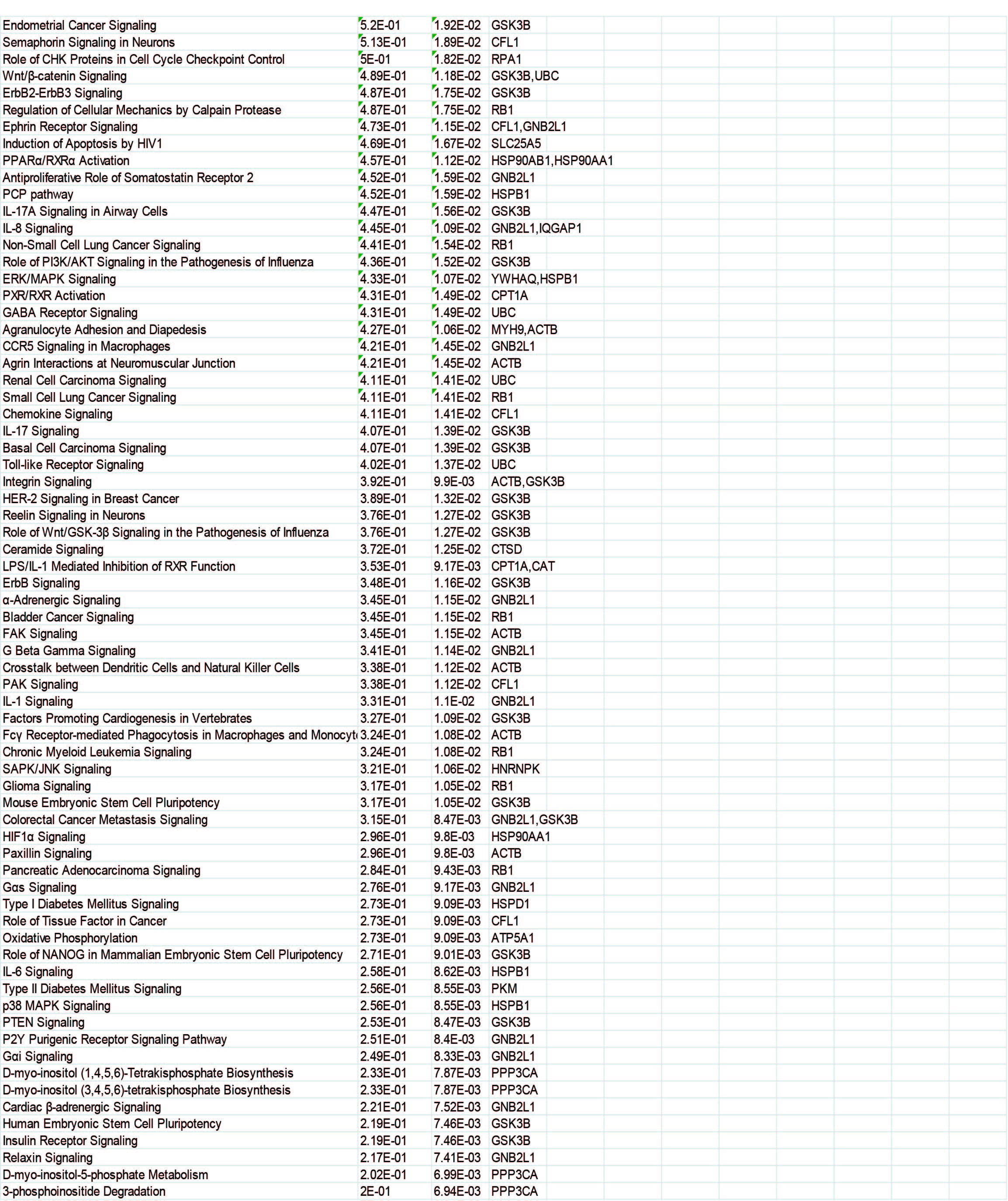
**

Supplement: Supplementary file 3 [file oncotarget-07-45715-s003.docx]
